# Anticancer Potential of Green Synthesized Silver Nanoparticles of the Soft Coral *Cladiella pachyclados* Supported by Network Pharmacology and In Silico Analyses

**DOI:** 10.3390/pharmaceutics13111846

**Published:** 2021-11-03

**Authors:** Hani A. Alhadrami, Heba Alkhatabi, Fahad H. Abduljabbar, Usama Ramadan Abdelmohsen, Ahmed M. Sayed

**Affiliations:** 1 Department of Medical Laboratory Technology, Faculty of Applied Medical Sciences, King Abdulaziz University, Jeddah 21589, Saudi Arabia; hanialhadrami@kau.edu.sa (H.A.A.); halkhattabi@kau.edu.sa (H.A.); 2 Molecular Diagnostic Lab., King Abdulaziz University Hospital, King Abdulaziz University, Jeddah 21589, Saudi Arabia; 3 Special Infectious Agent Unit, King Fahad Medical Research Center, King Abdulaziz University, Jeddah 21589, Saudi Arabia; 4 Center of Excellence in Genomic Medicine Research, King Abdulaziz University, Jeddah 21589, Saudi Arabia; 5 Department of Orthopedic Surgery, Faculty of Medicine, King Abdulaziz University, Jeddah 21589, Saudi Arabia; fhabduljabar@kau.edu.sa; 6 Department of Pharmacognosy, Faculty of Pharmacy, Minia University, Minia 61519, Egypt; 7 Department of Pharmacognosy, Faculty of Pharmacy, Deraya University, New Minia 61111, Egypt; 8 Department of Pharmacognosy, Faculty of Pharmacy, Nahda University, Beni-Suef 62513, Egypt

**Keywords:** *Cladiella*, silver nanoparticles, breast cancer, network pharmacology, in silico, docking, molecular dynamic simulation

## Abstract

*Cladiella*-derived natural products have shown promising anticancer properties against many human cancer cell lines. In the present investigation, we found that an ethyl acetate extract of *Cladiella pachyclados* (CE) collected from the Red Sea could inhibit the human breast cancer (BC) cells (MCF and MDA-MB-231) in vitro (IC_50_ 24.32 ± 1.1 and 9.55 ± 0.19 µg/mL, respectively). The subsequent incorporation of the *Cladiella* extract into the green synthesis of silver nanoparticles (AgNPs) resulted in significantly more activity against both cancer cell lines (IC_50_ 5.62 ± 0.89 and 1.72 ± 0.36, respectively); the efficacy was comparable to that of doxorubicin with much-enhanced selectivity. To explore the mode of action of this extract, various in silico and network-pharmacology-based analyses were performed in the light of the LC-HRESIMS-identified compounds in the CE extract. Firstly, using two independent machine-learning-based prediction software platforms, most of the identified compounds in CE were predicted to inhibit both MCF7 and MDA-MB-231. Moreover, they were predicted to have low toxicity towards normal cell lines. Secondly, approximately 242 BC-related molecular targets were collected from various databases and used to construct a protein–protein interaction (PPI) network, which revealed the most important molecular targets and signaling pathways in the pathogenesis of BC. All the identified compounds in the extract were then subjected to inverse docking against all proteins hosted in the Protein Data bank (PDB) to discover the BC-related proteins that these compounds can target. Approximately, 10.74% of the collected BC-related proteins were potential targets for 70% of the compounds identified in CE. Further validation of the docking results using molecular dynamic simulations (MDS) and binding free energy calculations revealed that only 2.47% of the collected BC-related proteins could be targeted by 30% of the CE-derived compounds. According to docking and MDS experiments, protein-pathway and compound-protein interaction networks were constructed to determine the signaling pathways that the CE compounds could influence. This paper highlights the potential of marine natural products as effective anticancer agents and reports the discovery of novel anti-breast cancer AgNPs.

## 1. Introduction

Green chemistry was developed as an effective alternative to other environmentally unsafe synthetic processes and related products [1,2]. In addition to its positive impact on the environment, it could result in a saving of about USD 65.5 billion per year by the end of 2021 [3]. Green methods for making metallic nanoparticles (MNPs) are preferred over chemical processes, as the latter have numerous negative environmental repercussions. Mixed-valence polyoxometalates [4], lower irradiation [5], polysaccharides [6], and biological methods [7,8] represent some of these environmentally friendly procedures. Biological methods are the most frequently used methods for the green preparation of MNPs [9]. In this approach, biological matrices are employed as reducing agents to synthesize MNPs and as a capping material to preserve them in a colloidal state [10]. Due to the surface-adsorbed molecules, this is an environmentally beneficial and cost-effective process that can add value to the manufactured MNPs [11]. Silver is one of the most studied metals for the preparation of MNPs, due to the availability of the starting material and the ease of preparation. Generally, AgNPs are well tolerated if used topically, while they have been reported to be associated with some toxicity when administered orally [12].

Marine settings have diverse ecosystems with a wide range of living forms, which can lead to unique chemical and biological features with significant therapeutic potential. Sponges are among the most promising marine species because of their unusually complicated chemistry and high biological activity. Similarly, soft corals are an interesting source of bioactive natural products, particularly diterpenes, triterpenes, and steroids. Anticancer properties against many cancer types are the most frequently reported bioactivities of soft-coral-derived natural products [13]. In comparison to terrestrial organisms, particularly plants, the capacity of marine organisms for preparing MNPs is less often studied [8]. We believe that biological matrices generated from marine species can synthesize MNPs by acting as reducing agents and enhance the therapeutic potential of the synthesized MNPs by adsorbing unique chemical entities onto the particle surface [12].

**Figure 1:**
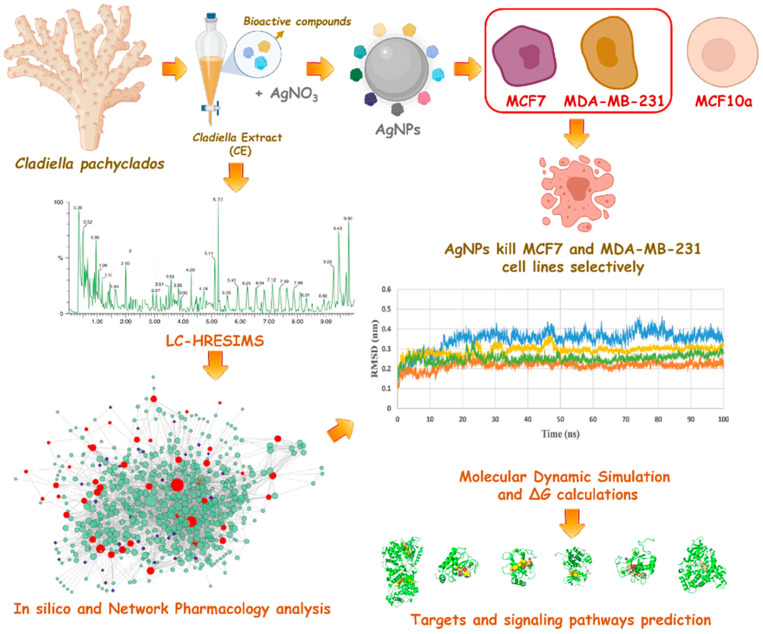
The applied strategy and the main findings of this study.

Herein, we investigated the potential of a crude organic extract produced from the marine soft coral *Cladiella pachyclados* to reduce aqueous AgNO_3_ to produce stable and bioactive silver nanoparticles (AgNPs). *C. pachyclados* has been reported to be a rich source of bioactive chemicals, particularly eunicellin diterpenoids which exhibit anticancer potential [14,15,16,17]. The anticancer efficacy of AgNPs produced by *Cladiella* extracts was then examined against two human breast cancer lines. Interestingly, this new nano-formulation was almost as effective as doxorubicin in killing breast cancer cells and was significantly more selective. To determine the possible mechanism of action of this bioactive extract, its LC-HRESIMS-annotated metabolites were subjected to comprehensive in silico and network-pharmacology-based investigations. This study could be an excellent starting point for a new anti-breast cancer formulation. It could help in the discovery of potent and specific anticancer chemical compounds. The outline of the present study is shown in Figure 1.

## 2. Materials and Methods

### 2.1. Collection of Marine Soft Coral

The marine soft coral (1.5 kg) was obtained in March 2021 at a depth of 7 m off the coast of Hurghada on the Red Sea (2,715,048″ north (N), 334,903″ east (E)). The Invertebrates Department, National Institute of Oceanography and Fisheries, Red Sea Branch, Hurghada, Egypt, was given a voucher sample (NIOF730/2021).

### 2.2. Preparation of the Organic Extract

Soft coral material was chopped into small pieces and extracted with ethyl acetate (EtOAc, 3 × 500 mL) using ultrasonic technology. Ethyl acetate was selected as a solvent because of its wide polarity and low toxicity. Accordingly, it can extract a wide range of natural products (polar and non-polar) without concerns about probable toxicity. A rotary evaporator (IKA^®^, Staufen, Germany) condensed the ethyl acetate extract (CE). The dried extract produced (95.23 g) was then stored at 4 °C prior to use.

### 2.3. LC-HRESIMS Chemical Profiling

The recovered extracts were subjected to chemical profiling using LC-HRESIMS according to our previous report [18]. The details of the LC-HRESIMS methodology are described in the Supplementary File (p. 19, Figures S1 and S2).

### 2.4. Preparation of Silver Nanoparticles

The green synthesis of silver nanoparticles was carried out according to Abdelhafez et al., 2020 [19]. Briefly, 0.005 g of crude extracts (CE) was dissolved in 1 mL dimethyl sulfoxide (DMSO), which acted as a co-solvent to make the CE miscible with aqueous solutions (e.g., AgNO_3_ solution). Then, 0.5 mL of this DMSO solution was mixed with 25 mL of 1 mM AgNO_3_. The mixture was stirred at 70 °C for 5 h in the dark until a change in the color of the mixture to yellowish brown indicated the reduction of AgNO_3_. The prepared AgNPs were kept in the dark for 60 days at 4 °C (no aggregations were detected after 60 days of storage).

### 2.5. Characterization of Silver Nanoparticles

#### 2.5.1. UV Spectroscopy

The synthesis of AgNPs was initially visualized by a color change in the solution. The transformation of Ag^1+^ to Ag^0^ was monitored by periodic sampling of aliquots (1 mL) of the mixture and measuring the UV–vis spectra of the solutions using a (SPECTROstar nano absorbance plate reader—BMG LABTECH). The UV–vis absorption spectra of the synthesized AgNPs remained almost identical over 60 days of storage (in the dark at 4 °C).

#### 2.5.2. X-ray Diffraction (XRD) Studies

The X-ray diffraction (XRD) patterns of the green prepared AgNPs were measured using a PANalytical X’pert PRO X-ray diffractometer with Cu Ka1 radiation under an operating voltage and a tube current of about 40 kV and 30 mA, respectively. The sample was drop-coated onto a glass substrate, and the X-ray diffraction patterns were recorded at values of 2θ from 10° to 80° with a scanning speed of 0.02°/min. XRD data were analyzed via JCPDS, now the International Centre for Diffraction Data, for the identification of the crystalline phases.

#### 2.5.3. Fourier Transform Infrared Spectroscopy (FTIR)

The ATR-FTIR spectra of the silver nanoparticles were acquired using a Bruker VERTEX 80 v spectrometer in the range 4000–400 cm^−1^ with a resolution of 4 cm^−1^.

#### 2.5.4. Transmission Electron Microscopy Analysis (TEM)

Transmission electron microscopy (TEM) was performed to examine the size and morphology of the synthesized silver nanoparticles. The sample preparation was carried out by placing 2–4 µL of either silver or gold nanoparticle solution on carbon-coated copper grids. The thin film formed was air-dried under ambient conditions and observed using a Philips 10 Technai TEM with an accelerating voltage of about 180 keV and a wavelength (λ) of 0.0251 Å. The average size of the prepared nanoparticles was measured using ImageJ software.

#### 2.5.5. Scanning Electron Microscope (SEM)

Scanning electron microscopy (SEM) was carried out using a field emission scanning electron microscope (FE-SEM) (Quanta FEG-250, Assen, The Netherlands) with an acceleration voltage of 20 kV, equipped with EDAX (energy-dispersive X-ray analysis) for the elemental analysis (Figure S3).

### 2.6. Antiproliferative Assay

The cytotoxicities of CE and the AgNPs were evaluated using an MTT assay (Promega, Dane County, WI, USA) according to the manufacturer’s protocol. Briefly, 5000 cells were plated in triplicates in the wells of a 96-well plate and incubated for 24 h at 37 °C in a 5% CO_2_ incubator (Thermo Fisher Scientific, Waltham, MA, USA). The cells were then treated with various concentrations of the test material or vehicle (PBS) for 24 h. After cell treatment, the MTT reagent was added to the cells and incubated for 4 h. The supernatant was aspirated, and 100 µL of DMSO was added to each well to dissolve the generated formazan crystals. The absorbances of the solutions were measured at a wavelength of 570 nm (630 nm was used as a reference filter). The cell viability was expressed as percentage inhibition relative to the vehicle-treated control. The nonlinear regression curve created by graphing the log concentration of the test material vs. %age inhibition yielded the IC_50_ (the concentration that caused a 50% inhibition of cell viability). The assay was performed in triplicates, and doxorubicin was used as a positive control. Further information can be found in the Supplementary File (pp. 9–18).

### 2.7. In Silico and Network Pharmacology Study

#### 2.7.1. In Silico ADME Profiling

The online software SwissADME (http://www.swissadme.ch/; accessed on 22 August 2021) was used to predict drug-like characteristics and the ADME profiles of all detected substances [20]. The ADME characteristics calculated included gastrointestinal (GI) absorption, blood–brain barrier (BBB), solubility, and bioavailability score. Additionally, the software calculates whether or not the query compounds follow both Lipinski’s and Veber’s rules [21,22].

#### 2.7.2. Anti-Breast Cancer Activity Predictions

Two independent machine-learning software platforms were used to predict the anti-breast cancer activity of LC-HRESIMS-identified compounds in CE. First, the compounds were submitted to pdCSM-cancer. This software utilizes a graph-based signature representation of the chemical structure of a huge number of small molecules to accurately predict candidates likely to be active against a single or multiple human cancer cell lines. After loading the query compounds, we selected breast cancer so that the software calculated the probable activity against most of the well-known breast cancer cell lines, including MCF7 and MDA-MB-231 (the two cell lines that were tested in vitro). The results indicate the following: (i) whether or not the compound has anticancer properties; (ii) whether or not the compound is active against the preselected cancer type, and which cell lines the query compound is predicted to be active against. According to the software algorithm, the compound is likely to be active against certain cancer cell lines if it has GI_50_% > 5 [23]. Secondly, the structures of the identified compounds were submitted to another neural-networking-based platform called CLC-Pred (http://www.way2drug.com/Cell-line; accessed on 25 August 2021). In this software, the results for the cellular toxicity predicted for each query compound were arranged according to a Pa score. The activity of a given compound can be predicted against many types of cancer and normal cell lines. A Pa score > 0.5 indicates a very high probability that the query compound is active [24].

#### 2.7.3. Target Proteins of Breast Cancer

BC-related target proteins (Table S1) were retrieved from the following four publicly available databases: GeneCards (https://www.genecards.org/; accessed on 22 August 2021) [25]; the Comparative Toxicogenomics Database (CTD, http://ctdbase.org/; accessed on 22 August 2021); the Therapeutic Target Database (TTD, http://db.idrblab.net/ttd/; accessed on 22 August 2021) [26]; the DrugBank database (https://www.drugbank.ca/; accessed on 22 August 2021) [27]. The words “Breast cancer, MCF7, and MDA-MB-231” were used as the keywords, and the species was set as “*Homo sapiens*”. The targets that occurred at least twice were chosen.

#### 2.7.4. Determination of the Potential Protein Targets of the Annotated Compounds

The potential protein targets for the CE-identified compounds were proposed by subjecting all of these compounds to inverse docking against all proteins hosted in the Protein Data Bank (PDB; https://www.rcsb.org/; accessed on 11 August 2021). The idTarget platform (http://idtarget.rcas.sinica.edu.tw/; accessed on 24 August 2021) was used for this task. This structure-based screening software applies a unique docking approach called divide-and-conquer docking that adaptively builds small overlapping grids to make the searching space on the protein surfaces more constrained. Hence, it can run a huge number of accurate docking experiments in a much-reduced time [28]. The retrieved results were obtained as a list of binding affinity scores arranged from the highest negative to the lowest. We set a binding affinity score of −7 kcal/mol as a cut-off value to select the best targets for each compound identified in CE. Considering the collected human breast cancer proteins, 26 protein targets were obtained for compounds **1**–**20** (Table S2).

#### 2.7.5. Molecular Dynamic Simulation and Binding Free Energy Calculation

The binding free energy calculation (∆*G*) and molecular dynamic simulation were carried out as previously described [18,29,30,31]. The Supplementary File has a detailed description of these methods (p. 19).

#### 2.7.6. Networks Construction and Functional Enrichment Analysis

We built three types of networks: (i) a network of compound-protein interactions (CPI) based on the prediction results. We built links between CE-derived compounds and BC target proteins in this network; (ii) a protein–protein interaction (PPI) network summarizing interactions between BC-relevant proteins. The proteins that were predicted to be probable targets for the CE compounds were submitted to the STRING database (https://string-db.org/; accessed on 25 August 2021) [32] for protein–protein interaction (PPI) analysis, where “Homo sapiens” was set as the search species, the interaction score was set to 0.9 (the maximum confidence), and the rest of the parameters were selected according to the default setting; (iii) a protein-pathway interaction network that showed the interactions between BC-related proteins and their associated signaling pathways. This network was constructed after KEGG pathway enrichment analysis. All the aforementioned networks were built using the Cytoscape 3.8.2 (https://www.cytoscape.org/; accessed on 29 August 2021) [33] network visualization and analysis software tool. The pre-installed tools carried out functional enrichment analysis and KEGG pathway (Figure S4) annotation for BC targets in Cytoscape 3.8.2.

### 2.8. Statistical Analysis

In this study, three independent experiments were conducted, with the results expressed as means and SEs (*n* = 3). ANOVA was used to establish statistical significance (*p* < 0.05).

## 3. Results

Natural compounds originating from marine organisms, particularly those derived from marine sponges and soft corals, are exceptionally efficient against human malignancies both in vitro and in vivo, and also during subsequent clinical studies. Furthermore, a number of effective anticancer medicines have been produced from such promising natural compounds, and they are presently utilized to treat cancer patients [34,35].

In our ongoing research into the discovery of possible natural-products-based anticancer therapeutics, we identified a *C. pachyclados*-derived EtOAc extract (CE) that exhibited promising antiproliferative activity against the human breast cancer estrogen-sensitive (MCF-7) and triple-negative (MDA-MB-231) cell lines (Table 1). Interestingly, this extract showed weak cytotoxicity towards normal breast cells (IC_50_ = 71.85 ± 3.57 µg/mL) and hence, good selectivity indices (SIs) for breast cancer cell lines that were comparable to those of the positive control doxorubicin (Table 1). Previously, *Cladiella*-derived natural products have exhibited antiproliferative potential against many human cancer cell lines, including breast cancer [14,15,16].

Accordingly, we used this bioactive extract as a reducing agent (i.e., CE) to prepare AgNPs, and hence, the final obtained nanoparticles were coated with CE. Thus, CE anticancer activity can be augmented with the aid of the unparalleled properties of the metallic nanoparticles (MNPs) in the following ways. (i) They can be utilized as an excellent carrier for bioactive matrices including natural-products-based extracts, (ii) the natural-products-coated MNPs can easily penetrate cellular membranes [29,36,37,38], and (iii) MNPs have a unique ability to accumulate and concentrate bioactive chemical compounds on their surfaces and thus maximize their biological activity (polyvalent action) [39,40,41,42,43]. Moreover, the SI values of these newly prepared AgNPs were significantly higher than those of the standard anti-breast cancer agent, doxorubicin. More details on the antiproliferative activity can be found in the Supplementary File (pp. 9–18, Figures S5 and S6).

The cellular toxicity of the prepared AgNPs against normal breast cancer was fairly high (IC_50_ = 41.29 ± 0.51 µg/mL), which could be attributed to the slowly released silver ions [17]. However, the safety index is comparable to that of doxorubicin.

### 3.1. CE-Mediated Green Biosynthesis of Silver Nanoparticles

**Figure 2:**
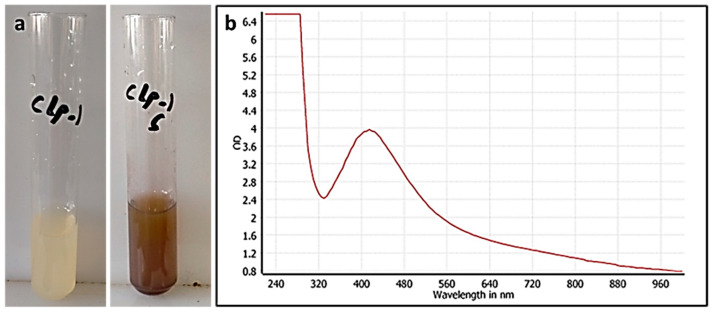
(**a**) Color change due to AgNPs, (**b**) UV–vis spectrum after synthesis of AgNPs.

AgNPs were green synthesized using CE as a reducing agent. Typically, a change in color to a yellowish-brown was seen in the mixture after metal-ion reduction, indicating the reaction with the Ag^+^ ions and the formation of silver nanoparticles (i.e., Ag^0^ NPs) (Figure 2a). A yellow-brown color formed due to the excitation of surface plasmon vibrations in the particles was detected.

### 3.2. Characterization of the Prepared AgNPs

#### 3.2.1. UV Spectroscopy

The formation of AgNPs was confirmed by UV–vis absorption spectroscopy (Figure 2b). The appearance of the absorbance band at 420 nm indicates the formation of AgNPs. These results were in agreement with previous studies [44,45]. The stability of the prepared AgNPs was monitored over 60 days (at ~25 °C). The UV–vis absorption spectra of the green synthesized AgNPs remained almost identical until the last day of the study (Figure S7). For the in vitro anticancer study, three CE-AgNPs samples were taken: after 1 day, 30 days, and 60 days from preparation.

#### 3.2.2. Electron Microscopy

**Figure 3:**
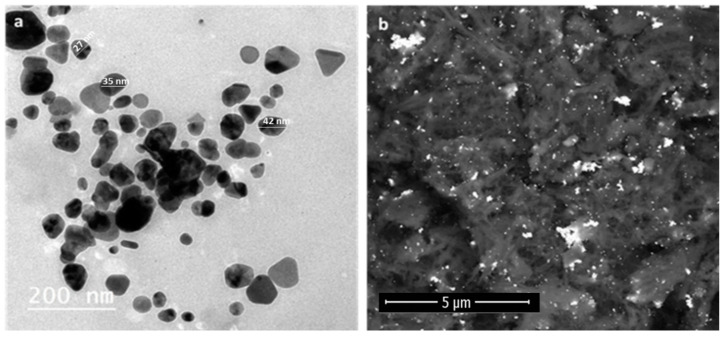
(**a**) TEM micrographs for poly-dispersed shapes and triangle-shaped CE-AgNPs; (**b**) FE-SEM micrographs for prepared CE-AgNPs.

The particle size and morphology of the prepared AgNPs were measured using transmission electron microscopy (TEM) and field emission scanning electron microscopy (FE-SEM) Figure 3. The average particle size for the CE-AgNPs was about ~3 ± 2 to 25 ± 2 nm with poly-dispersed shapes and triangle-shaped AgNPs (Figure 3a,b).

#### 3.2.3. X-ray Powder Diffraction (XRD)

**Figure 4:**
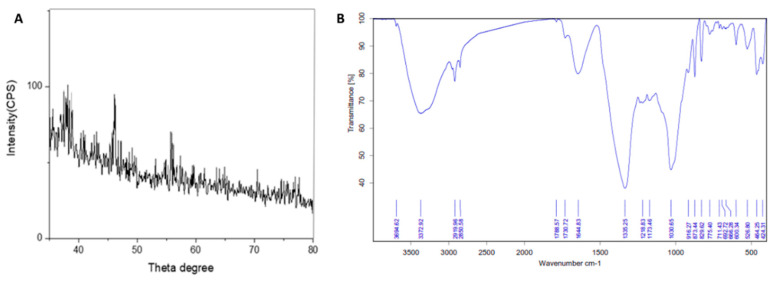
(**A**) X-ray diffraction pattern of the AgNPs; (**B**) FTIR spectra of the prepared AgNPs.

An X-ray diffraction (XRD) analysis was carried out to study the structural properties of the green synthesized AgNPs. The XRD results for the AgNPs showed that the essential characteristic peaks of the Ag phase appear at 38.00°, 45.01°, and 57.62°, attributed to the crystallographic planes (111), (200), and (220) of silver (Figure 4A).

#### 3.2.4. Fourier Transform Infrared Spectroscopy Analysis (FTIR)

The FTIR analysis was performed to identify the functional groups responsible for the synthesis and stabilization of silver nanoparticles. The FTIR spectral profile of the green AgNPs showed peaks at 3372.92, 2919.92, 2850.58, 1788.57, 1730.72, 1335.25, 1218.83, and 1030.65 cm^−1^, corresponding to different functional groups, namely, OH, CH, C=O, C=C, C-O, C-N, and C-C in the prepared nanoparticles (Figure 4B). The hydroxyl group (O-H) stretching vibration appears at 3372.92, while peaks at 2919.92 and 2850.58 cm^−1^ refer to the alkane (C-H) stretching vibration. Moreover, the carbonyl (C=O) stretching vibration occurred at 1788.57 cm^−1^. The aromatic (C=C) stretching bands were observed at 1730.72 cm^−1^. C-O stretching was measured at 1335.25, and the band at 1218.83 cm^−1^ refers to C-N stretching. Additionally, the C-C stretching appears at 1030.65 cm^−1^. The presence of an absorption band corresponding to C=C, C=O, C-O, C-N, OH, and CH could be mainly attributed to the sterols, diterpenes, and alkaloids that were found to be major constituents in CE (Table 2). These functional groups play a specific role in the reduction of Ag metal ions and the stability of the prepared AgNPs.

### 3.3. LC-HRESIMS-Assisted Chemical Profiling of CE

**Figure 5:**
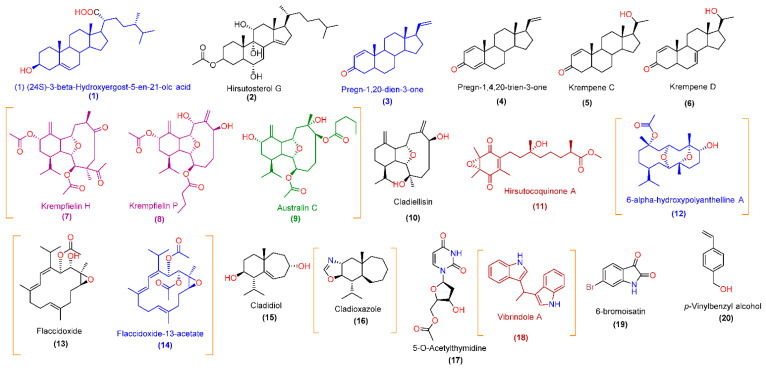
Chemical structures of major compounds **1**–**20** (peak area > 10,000) that have been annotated in CE. According to the pdCSM-cancer software, blue-colored compounds (**1**, **3**, **12**, and **14**) were predicted to have general anticancer activity; purple-colored compounds (**7** and **8**) were predicted to have anticancer activity against MCF7 and MDA-MB-231 cell lines; brown-colored compounds (**11** and **18**) were predicted to have anticancer activity against MCF7 only. The green-colored compound (**9**) was predicted to have general anticancer activity along with specific anticancer activity towards MCF7 and MDA-MB-231 cell lines. According to the CLC-Pred software, compounds between orange parentheses were predicted to be active (Pa > 0.5) against either MCF7 (compounds **7**–**9** and **16**) or MDA-MB-231 cell lines (compounds **12**–**14** and **18**). Black-colored compounds were inactive according to both prediction software platforms.

LC-HRESIMS-based chemical characterization of CE led to the annotation of 20 major compounds in the extract (Table 2, Figure 5). The dereplicated compounds (**1**–**20**) were found to belong to three main classes of natural products (i.e., sterols, terpenoids, and alkaloids). All 20 annotated compounds have been previously reported in different *Cladiella* sp. [14,15,16,46,47,48,49,50,51,52]. Eunicellin-type (**7**–**10**) and cembrane-type (**12**–**14**) diterpenes are very characteristic of *Cladiella* soft coral and have demonstrated potent antiproliferative effects against many types of human cancer cell lines [51,52].

Vibrindole A and 6-bromoisatine (compounds **18** and **19**, respectively) have been isolated from many other marine organisms and microorganisms and exhibit good activity against many human cancer cell lines [53,54,55,56,57,58]. The conjugation of such a bioactive extract rich in a variety of interesting bioactive chemical entities to MNPs such as silver has great potential to augment its biological effects, particularly the antiproliferative effects.

### 3.4. In Silico Investigation

With the aid of computer software and modern algorithms (e.g., artificial intelligence and machine learning), biological activity prediction has become widely utilized as an integral tool in the drug discovery process. Such computer-aided and virtual (i.e., in silico) techniques could also be used in natural-products-based drug discovery, where they can annotate and characterize the most probable active metabolite from a complex mixture (i.e., crude extract) [59,60,61], propose the mode of action, or suggest potential molecular targets for the major compounds in a given natural-products-based extract [62,63,64,65,66,67].

Accordingly, we subjected the identified metabolites (**1**–**20**) in the CE to several machine learning and neural-networking-based prediction programs to assign the main compounds responsible for the anticancer activity of CE. Moreover, the molecular targets of these compounds can be proposed with the aid of this type of predictive software. Hence, networks between the compounds and their targets and between the targets themselves can be constructed to obtain some insight into the main pathways that could be influenced by these extract-derived components (i.e., network pharmacology) [67,68,69].

#### 3.4.1. CE-Derived Compounds with Proposed Antiproliferative Activity against BC Cell Lines

**Figure 6:**
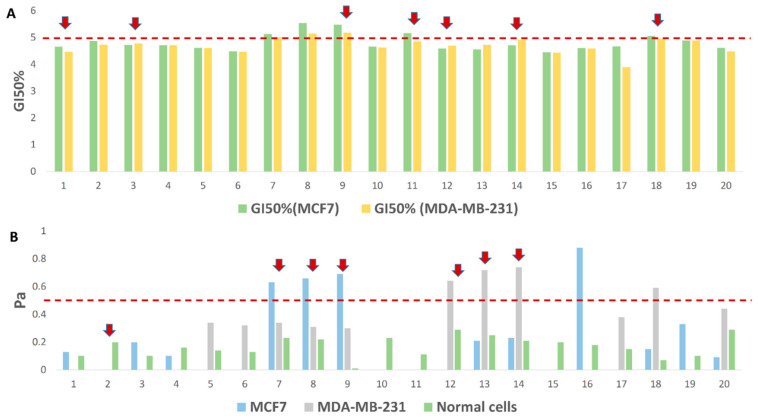
(**A**) The pdCSM-cancer prediction scores of compounds **1**–**20** as anticancer agents. Compounds with GI50% > 5 are probably active against either MCF7 and/or MDA-MA-231 cell lines. Red arrows point to compounds that were predicted to be generally active as anticancer agents. (**B**) The CLC-Pred prediction scores of compounds **1**–**20** as anticancer agents. Pa > 0.5 indicates high probability of activity against either MCF7 and/or MDA-MA-231 cell lines in vitro. Red arrows point to compounds that were predicted to be active against other cancer cell lines in the software’s database.

First, we subjected the dereplicated compounds (**1**–**20**) in CE to two independent prediction software platforms utilizing neural-networking-based algorithms, namely, pdCSM-cancer and CLC-Pred. The former utilizes a graph-based signature representation of the chemical structure of a huge number of small molecules to precisely predict candidates likely to be active against single or multiple human cancer cell lines. This recently reported predictive software is the most comprehensive anticancer bioactivity prediction platform established to date, consisting of trained and validated models of experimental data on the growth inhibition concentration (GI_50_%) effects of approximately 18,000 compounds on 74 distinct cancer cell lines representing nine tumor types. Across 10-fold cross-validation, it achieved Pearson’s correlation coefficients of up to 0.74. According to pdCSM-cancer predictions, compounds **7**–**9** were probably active against both MCF7 and MDA-MB-231 cell lines (GI_50_% > 5) (Figure 5 and Figure 6), while compounds **11** and **18** were probably active against MCF7 only. In addition to its ability to predict the activity towards certain cell lines, the developed algorithm of this software can also recognize the general potential of specific chemical motifs to have anticancer properties. For instance, compounds **1**, **3**, **9**, **12**, and **14** were predicted to have general anticancer activity (Figure 5 and Figure 6).

On the other hand, CLC-Pred (Cell Line Cytotoxicity Predictor) software utilizes a neural-networking-based prediction algorithm. Compounds that score Pa > 0.5 according to PASS in biological activities are probably active upon experimental validation, and those with Pa < 0.5 are not. Accordingly, compounds **7**–**9** and **16** were predicted to be active against the MCF7 cell line, while compounds **12**–**14** and **18** were predicted to be active against MDA-MB-231 (these compounds had Pa scores > 0.5). The remaining compounds had Pa scores < 0.5 or were predicted to be active against other cancer cell lines (Figure 5 and Figure 6). It is worth noting that all compounds (**1**–**20**) were probably non-toxic to the normal cell lines hosted in the database of this prediction platform (Pa < 0.5).

According to the prediction results of the two software platforms, compounds **7**–**9** and **18** were the most probable bioactive metabolites in CE. Interestingly, most of the predicted compounds have been reported to exert significant growth inhibitory activity against either MCF7, MDA-MB-231, or other cancer cell lines [14,15,16,17,46,47,48,49,50,51,52].

#### 3.4.2. Protein Targets Associated with BC and the Interactions between Them (PPI Network)

**Figure 7:**
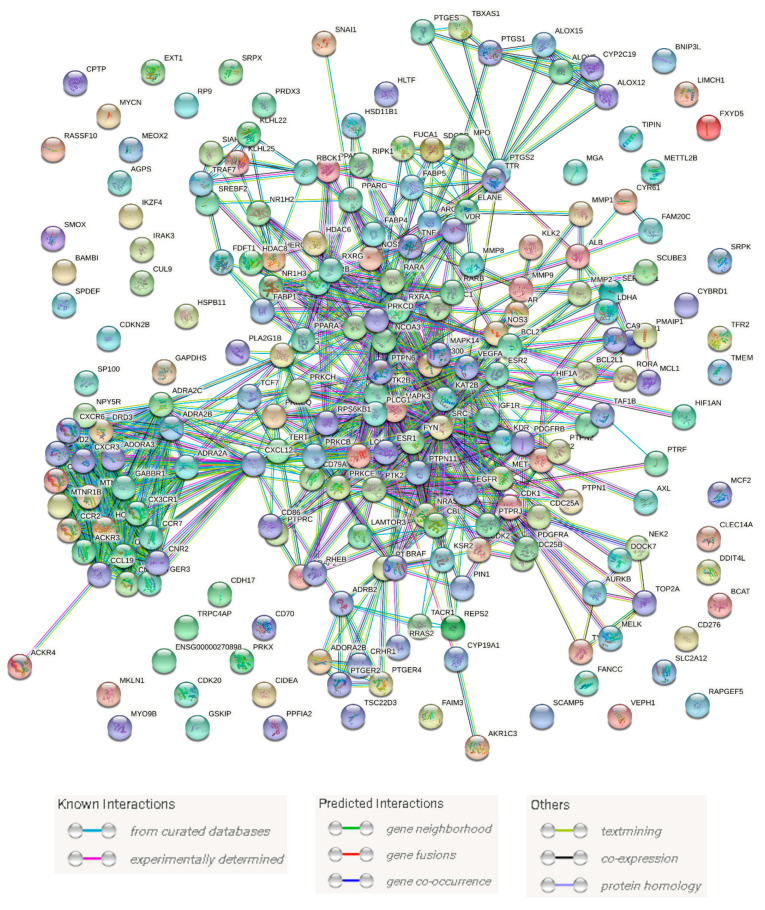
BC PPI network. This network consists of 214 nodes and 491 edges with an average node degree of 4.75.

**Figure 8:**
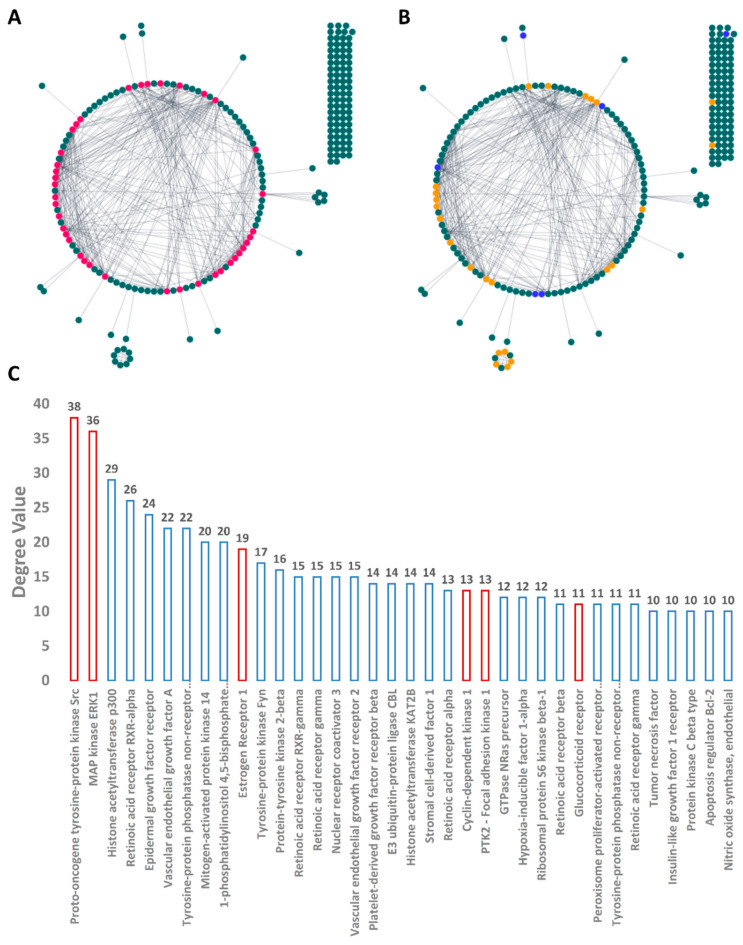
BC PPI networks showing the top 15% of nodes (i.e., hub proteins; red-colored nodes): (**A**) the proteins that were predicted to be targeted by CE-derived compounds (**1**–**20**; orange and blue-colored nodes); (**B**) blue nodes represent the proteins that were validated by MDS and Δ*G* calculation as targets for CE-derived compounds (**1**–**20**); (**C**) the top 15% nodes (i.e., hub nodes) arranged by their degree value. Orange-colored columns represent the proteins found to be among the predicted targets for CE-derived compounds (**1**–**20**).

To obtain information about all proteins or genes linked to human BC, we utilized two publicly available databases: GeneCards and the Toxicogenomics Database. Additionally, we obtained further information about genes or proteins that have been reported to have a direct link with multidrug-resistant and triple-negative BC in the literature [70,71]. As a result, we collected 242 genes or proteins related to BC (i.e., MCF7 and MDA-MA-231 cell lines). Subsequently, these proteins were utilized to construct a protein–protein network (PPI) with the aid of the STRING database. As shown in Figure 7, the produced PPI network showed many interactions composed of 491 edges between 214 nodes with an average node degree of 4.75. Highly interacting proteins are considered the most relevant and essential targets in BC, and thus targeting such proteins significantly increases the success rate against this disease. Accordingly, we extracted the top 15% (36 proteins) of highly interacting nodes (i.e., hub proteins) ordered by their degree value (Figure 8).

#### 3.4.3. Predicted Targets for the Active Chemical Compounds in CE

**Figure 9:**
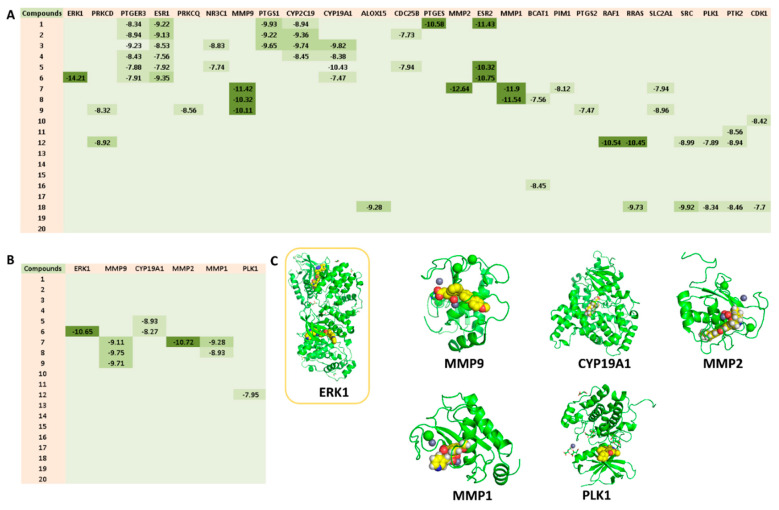
(**A**) Heat map showing docking scores in kcal/mol of compounds **1**–**20** with 26 BC-related proteins. A cut-off of −7 kcal/mol was set to select the top-scoring BC-related protein targets for compounds **1**–**20**. (**B**) Heat map showing MDS-derived Δ*G* values for a number of CE-derived compounds inside the binding sites of 6 out of 26 protein targets (**C**) predicted to be potential BC targets. ERK1 (the protein structure inside a yellow rectangle) is one of the highly interacting proteins (i.e., hub proteins) in the BC PPI network.

**Figure 10:**
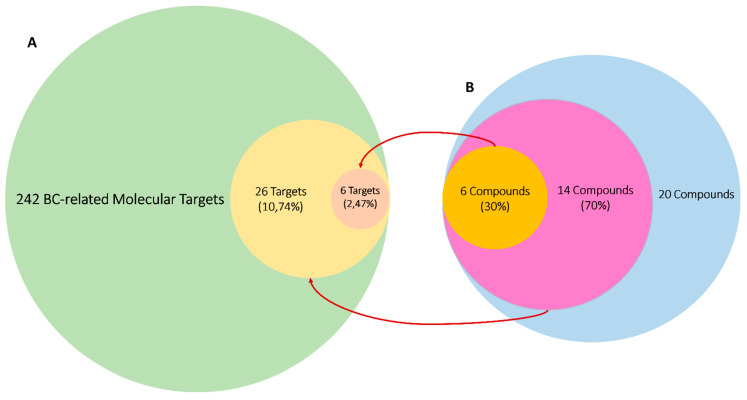
(**A**) The green-colored circle indicates all collected BC-related molecular targets, the yellow-colored circle indicates BC-related targets that were found to be potential targets for CE-derived compounds, and the brown-colored circle indicates protein targets that formed stable complexes with 6 CE-derived compounds over 100 ns of MDS. (**B**) The blue-colored circle indicates all major compounds that were annotated in CE (**1**–**20**), the pink-colored circle indicates compounds that obtained docking scores < −7 kcal/mol with one or more BC-related proteins, and the orange-colored circle indicates compounds that achieved stable binding with their corresponding proteins. Red arrows indicate interactions between different circles.

According to Lipinski’s and Veber’s drug-likeness rules, all compounds annotated in CE were drug-like compounds. Hence, we subjected compounds **1**–**20** to an inverse docking approach using the idTarget docking platform to accurately propose the most probable molecular targets of these compounds (**1**–**20**). The idTarget platform applies a unique docking approach called divide-and-conquer docking that adaptively builds small overlapping grids to make the searching space on the protein surfaces more constrained. Hence, it can run a huge number of accurate docking experiments in a much-reduced time. This efficient software can dock the query compound against almost all protein structures hosted in the protein data bank (PDB). The retrieved results were obtained as a list of binding affinity scores arranged from the highest negative value to the lowest. We set a binding affinity score of −7 kcal/mol as a cut-off value to select the best targets for each identified compound in CE. Considering the collected human breast cancer proteins, 26 protein targets were obtained for compounds **1**–**20** (Table S2). The binding affinity scores of each compound with its corresponding proteins are summarized in Figure 9. For further validation, we subjected each retrieved compound-protein complex to a 100 ns MDS experiment to study the stability of each compound inside the active site of each corresponding protein and to calculate its binding free energy (Δ*G*). In this step, we set a Δ*G* value of −7 kcal/mol as a cut-off to select the best ligands (i.e., inhibitors). Additionally, the compound should also be stable inside the binding site of its corresponding protein during the MDS (i.e., obtain an average RMSD < 5 Å) to be selected. As a result, only six proteins were highly probable targets for compounds **3**, **4**, **6**–**9**, and **12** (Figure 9). Interestingly, 6 out of the 26 predicted proteins were among the top-interacting (hub) proteins in the BC network (Figure 8, Figure 9 and Figure 10). MAP kinase ERK1 was the only hub protein whose binding with compound 6 was stable over the course of 100 ns of MDS. These findings highlighted that major compounds in CE are likely to act on BC cell lines by targeting multiple key proteins. The results of the target proteins prediction were also in good accordance with those of pdCSM-cancer and CLC-Pred (Figure 7), particularly for compounds **7**–**9,** which were common hits in all previous experiments.

#### 3.4.4. PPI and CPI Networks of the Predicted Targets and KEGG Enrichment Analysis

**Figure 11:**
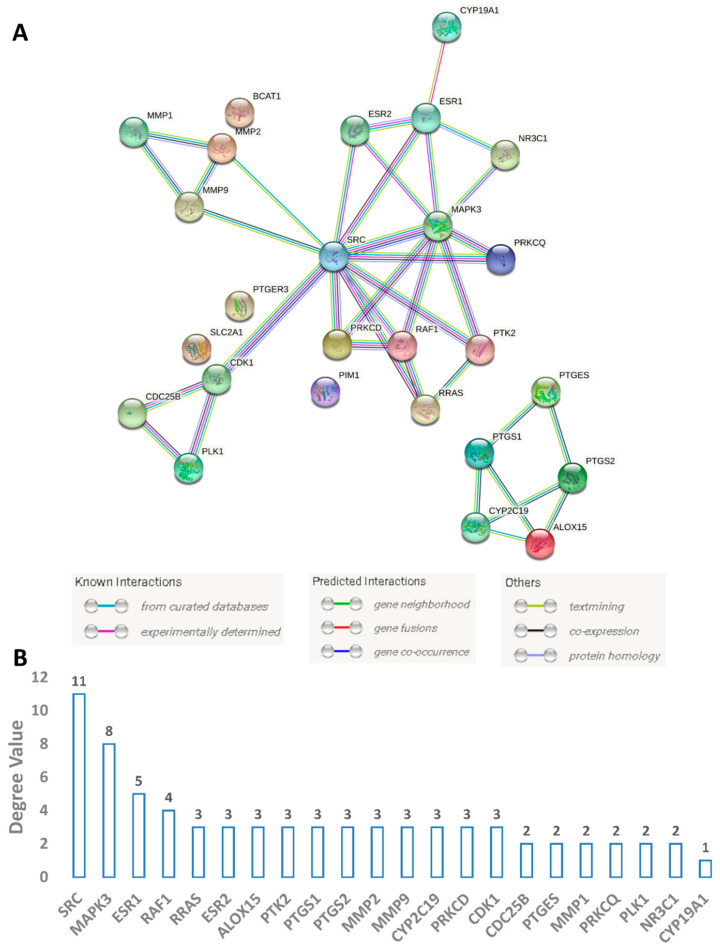
(**A**) BC sub-PPI network constructed from proteins predicted to be targeted by CE-derived compounds **1**–**20**. This network consists of 26 nodes and 37 edges with an average node degree of 2.85. (**B**) Degree values of the interacting nodes.

Protein targets predicted for CE-derived compounds (**1**–**20**) were then used to construct a sub-PPI network (Figure 11) and a KEGG enrichment analysis to explore the mode of action of CE. As shown in Figure 11, all the predicted proteins were connected (average node degree 2.85) except for BCAT1, SLC2A1, PIM1, and PTGER3. SRC and MAPK3 (also known as ERK1) (Proto-oncogene tyrosine-protein kinase Src and MAP kinase ERK1, respectively) were the most highly interacting nodes and were located in the center of the network. Both proteins are key protein kinases in BC and have been studied extensively for targeting by small-molecule inhibitors [72,73].

**Figure 12:**
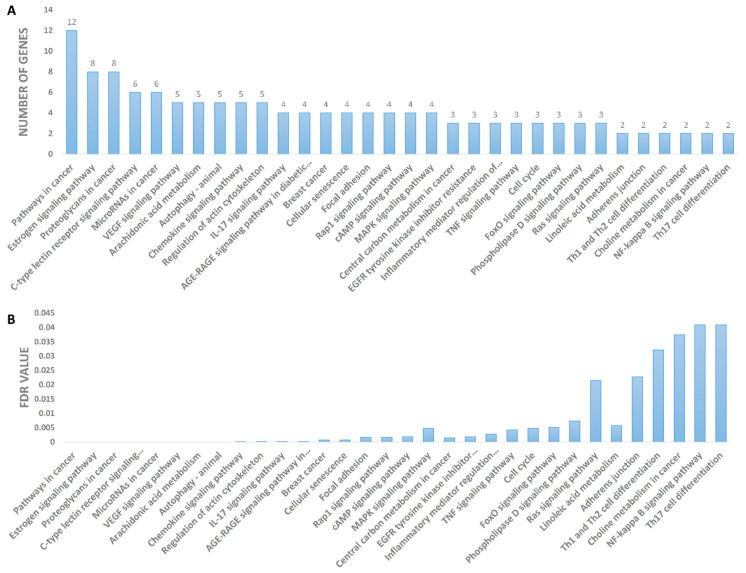
KEGG pathway enrichment. (**A**) *X*-axis is enrichment gene count, *Y*-axis is KEGG pathway. (**B**) False discovery rate (FDR) of each annotated pathway.

**Figure 13:**
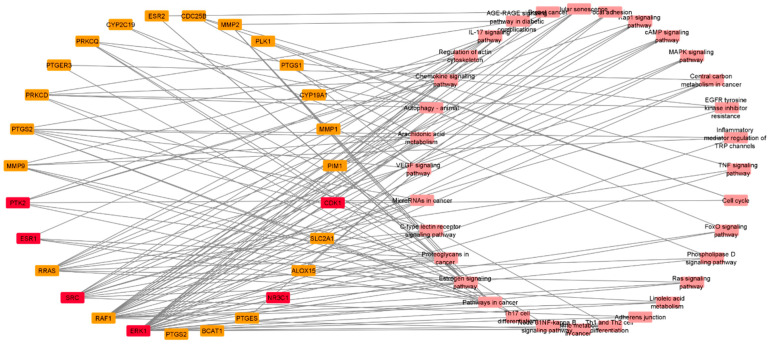
Protein-pathway network. This network reveals the most important genes or proteins in the enriched KEGG pathways. Targets in red squares were identified as highly interacting proteins (i.e., hub proteins) in the BC PPI network.

The KEGG pathway analysis (i.e., enrichment analysis) was used to determine the most important signaling pathways associated with the anti-breast cancer activity of CE. As revealed in Figure 12, pathways in cancer, estrogen signaling, proteoglycans, c-type lectin receptor signaling, microRNAs in cancer, VEGF signaling, arachidonic acid metabolism, autophagy, breast cancer, and cellular senescence are among the top signaling pathways that were proposed as being influenced by CE compounds (i.e., having the highest number of proteins targeted by CE compounds). Significantly enriched genes are described in detail in the Supplementary Materials (Table S3). A protein-pathway network was constructed based on the KEGG enrichment analysis (Figure 13), highlighting SRC, MAPK3, and ESR1 as the most enriched genes.

**Figure 14:**
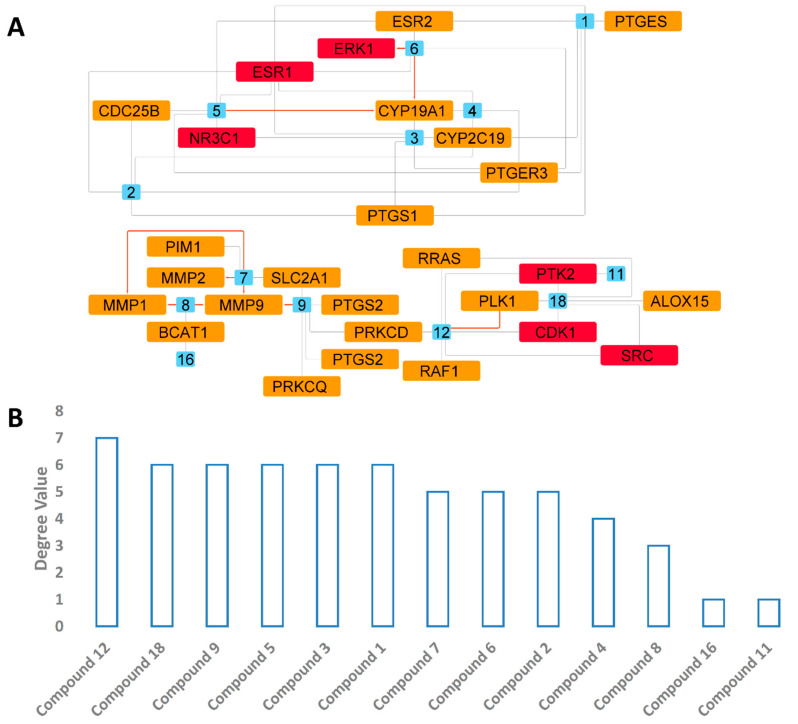
Compound-protein network. (**A**) This network consists of 39 nodes and 98 edges with average node degree of 4.23. Blue numbered nodes represent CE-derived compounds, and orange nodes represent BC-related proteins. Red nodes are hub proteins in the BC PPI network. (**B**) Degree values of the interacting compounds. Red-colored edges indicate MDS-validated interactions.

A compound-protein network was constructed based on the docking results that revealed the potential BC-related protein targets for the CE compounds (**1**–**20**) (Figure 14). Only 13 out of 20 compounds showed interactions with a number of BC-related proteins (Figure 10). Most of these interacting compounds showed interactions with ~6 different proteins. Compound **12** was the most interacting compound (7 interactions), and compound **11** was the least (1 interaction). It is worth noting that MDS-validated interactions have greater confidence (red-colored edges; Figure 14), and hence, these interactions will be further analyzed by molecular modelling in the next section. According to the previous in silico findings, CE could inhibit the growth of both MCF7 and MDA-MA-231 via many cancer-related pathways and via interaction with many oncoproteins. Incorporating this rich bioactive extract onto AgNPs made its bioactive component more concentrated (polyvalent effect), and thus the whole formulation became much more potent against both BC cell lines with excellent selectivity that was even better than that of the reference anti-breast cancer drug, doxorubicin.

#### 3.4.5. Molecular Modeling

Inverse molecular docking experiments revealed that most CE-derived compounds (**1**–**20**) could achieve good binding with a number of BC-related proteins (Figure 9 and Figure 10). Upon further validation of their binding stability via MDS, only six compounds were found to maintain their binding orientations inside the corresponding binding sites over 100 ns of MDS. Additionally, they had the highest Δ*G* values, indicating that they could be considered as potential binders (i.e., inhibitors) for many key BC proteins.

**Figure 15:**
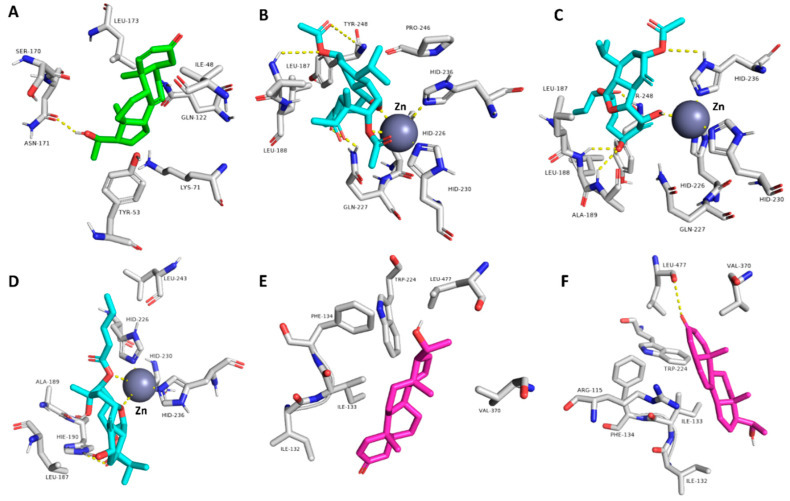
(**A**) Binding mode of compound **6** inside the active site of MAP kinase ERK1 (ERK1; green-colored compound); (**B**–**D**) binding modes of compounds **7**–**9**, respectively, inside the active site of matrix metalloproteinase-9 (MMP9; cyan-colored compounds); (**E**,**F**) binding modes of compounds **5** and **6,** respectively, inside the active site of cytochrome P450 19A1 (aromatase, CYP19A1; pink-colored compounds). All of these binding modes were the last snapshot derived from MDS of each compound-protein complex.

**Figure 16:**
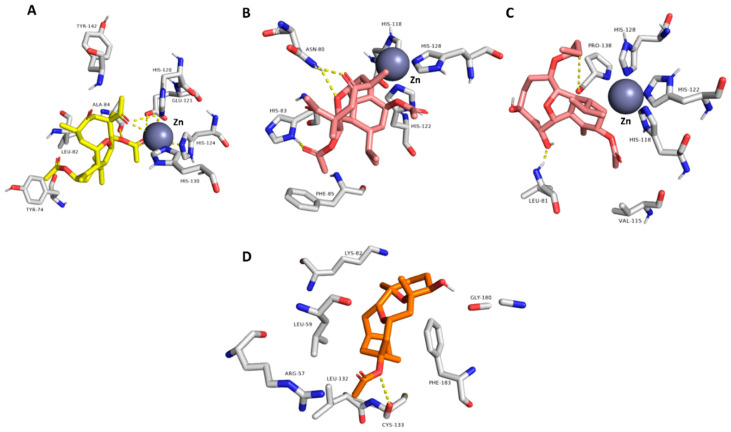
(**A**) Binding mode of compound **7** inside the active site of matrix metalloproteinase-2 (MMP2; yellow-colored compound); (**B**,**C**) binding modes of compounds **7** and **8**, respectively, inside the active site of matrix metalloproteinase-1 (MMP1; red-colored compounds); (**D**) binding mode of compound **12** inside the active site of serine/threonine-protein kinase PLK1 (PLK1; orange-colored compound). All of these binding modes were the last snapshot derived from MDS of each compound-protein complex.

**Figure 17:**
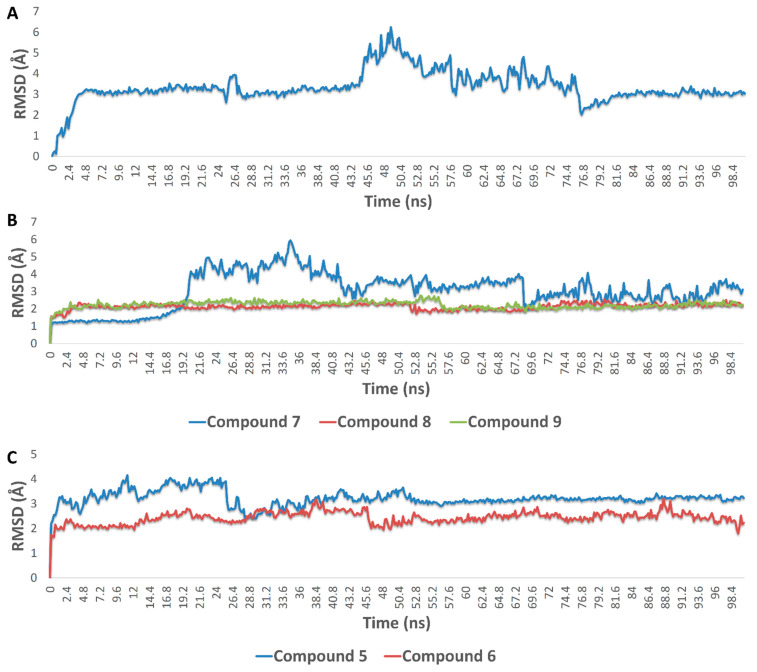
(**A**) RMSDs of compound **6** inside the active site of MAP kinase ERK1 (ERK1) over 100 ns MDS; (**B**) RMSDs of compounds **7**–**9** inside the active site of matrix metalloproteinase-9 (MMP9) over 100 ns MDS; (**C**) RMSDs of **5** and **6** inside the active site of cytochrome P450 19A1 (aromatase, CYP19A1) over 100 ns MDS.

**Figure 18:**
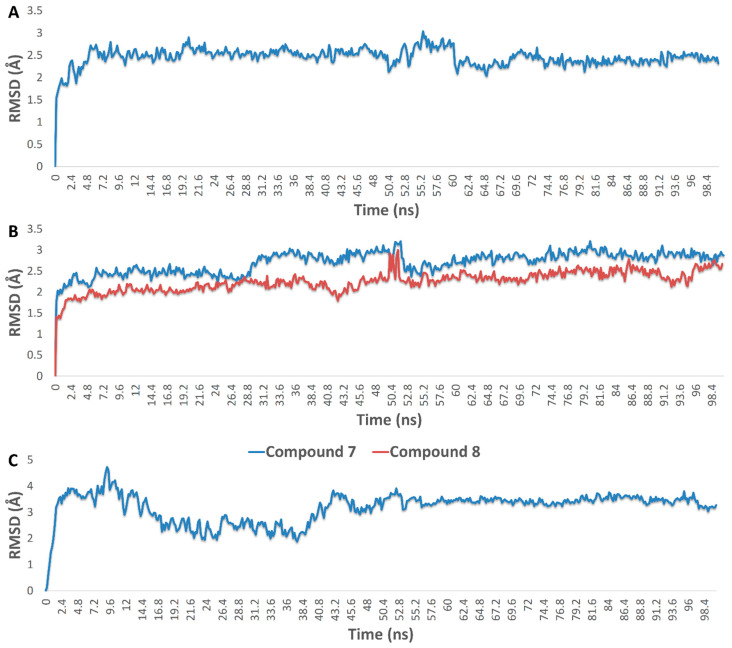
(**A**) RMSDs of compound **7** inside the active site of matrix metalloproteinase-2 (MMP2) over 100 ns MDS; (**B**) binding modes of compounds **7** and **8** inside the active site of matrix metalloproteinase-1 (MMP1) over 100 ns MDS; (**C**) binding mode of compound **12** inside the active sites of serine/threonine-protein kinase PLK1 (PLK1) over 100 ns MDS.

Accordingly, the binding modes of these compounds (**5**–**9** and **12**) were investigated to determine how they can interact inside each binding site of their corresponding protein targets. The compound **6** (krempene D) binding mode inside the active site of MAP kinase ERK1 (ERK1) was stabilized over 100 ns of MDS (average RMSD = 3.2 Å) via four hydrophobic interactions with ILE-48, TYR-53, LYS-71, and LEU-173, and single strong H-bond (<2.5 Å) with ASN-171 (Figure 15, Figure 16, Figure 17 and Figure 18). Consequently, it had a Δ*G* value of −10.65 kcal/mol. Docking of compound **5** (krempene C), the congener of compound **6**, inside the active site of ERK1 resulted in a binding mode convergent to that of compound **6**. However, it could not remain inside the active site up to the end of MDS (average RMSD = 15.8 Å) with a significantly high Δ*G* value (−2.2 kcal/mol). ERK1 is one of the key oncoproteins that regulate the development of BC and was found to be linked to a poor prognosis and poor response to hormonal therapy [74,75,76]. Several small molecules that were found to interfere with the function of this protein have shown very good efficacy against several types of BC [77,78,79].

Eunicellin-type diterpenes **7**–**9** (krempfielin H and P and australin C) are among the characteristic metabolites in many *Cladiella* sp. These unique compounds were found to interact with several human matrix metalloproteinases (MMP1, 2, and 9). Compound **7** established stable binding with the three proteins, while compound **8** interacted with MMP1 and 9, and compound **9** with MMP9 only (Figure 15, Figure 16, Figure 17 and Figure 18). H-bonding governed the interactions and, in turn, the stability of the three compounds inside the active site of MMP9 (Figure 15). In addition, they were able to establish co-ordinate interactions with Zn^+2^ inside the enzyme’s active site. Their binding orientations remained stable up to the end of MDS with very slight deviations (RMSD~3.5 Å) from their original docking-derived binding modes (Figure 16 and Figure 17). Their Δ*G* values were also convergent (~−9.5 kcal/mol; Figure 9). The binding mode and Δ*G* value of compound **7** with both MMP1 and 2, and of compound **8** with MMP1, were comparable to those of MMP9 except for the interactions with the active site’s Zn^+2^ (Figure 17). Human matrix metalloproteinases (MMPs) are types of hydrolases that are overexpressed in different types of human cancers including BC [80,81,82], but their exact role in tumor development and progression is still elusive [83,84,85]. Several MMP-specific inhibitors have been found to significantly inhibit the proliferation of many cancer types, particularly MMP9, for which inhibition has been associated with promising outcomes in BC [86].

Human aromatase (HA; CYP19A1) is a key enzyme involved in the biosynthetic pathway of estrogen. Accordingly, scaffolds similar to estrogen can easily access the active site of this enzyme and, in turn, inhibit its function. Compounds **5** and **6** (krempene C and D) are pregnan-type compounds, and their structures are very close to that of estrogen. Their binding mode inside HA’s active site is mainly governed by hydrophobic interactions (with ILE-132, ILE-133, PHE-134, TRP-224, LEU-447, and VAL-370), just as for estrogen. Compound **6** established an additional H-bond with Leu-477 (Figure 15). These binding orientations remained unchanged over 100 ns of MDS with low deviations from the original starting orientation (RMSD~2.3 Å) (Figure 17). Moreover, they had low Δ*G* values of ~−8.5 kcal/mol, indicating a good binding affinity to the enzyme active site. Compounds **3** and **4** (pregn-1,20-dien-3-one and pregn-1,4,20-trien-3-one, respectively) had structures and binding scores similar to those of compounds **5** and **6**. However, they had significantly high Δ*G* values (~−4.7 kcal/mol), and hence, their estimated binding affinity was lower than that of compounds **5** and **6**. HA is overexpressed in BC to make cancer cells produce more estrogen, which in turn increases tumor proliferation and development [87]. Hence, HA inhibitors are now used alongside other BC therapeutics to treat or prevent tumor recurrence [88,89].

Finally, compound **12** (6-alpha-hydroxypolyanthelline A) was able to establish a stable binding with focal adhesion kinase 1 (PTK2) over 100 ns of MDS (RMSD~3 Å). Accordingly, it had the best Δ*G* value (−7.95 kcal/mol) with this BC-related protein. Its binding mode with the enzyme’s active site was mediated mainly by a strong H-bond with CYS-133 (<2.5 Å) and three hydrophobic interactions with Leu-59, LYS-82, and PHE-183 (Figure 16). PTK2 is involved in cellular adhesion and spreading processes. It has been shown that the inhibition of this functional enzyme makes breast cancer cells less metastatic due to decreased mobility [90,91,92].

## 4. Discussion

Natural-product-based drug discovery has gained increased attention in recent years. Additionally, the continuous and rapid development of in silico drug discovery approaches have made it easy to explore the therapeutic potential of natural products, particularly those that occur as a complex mixture of chemical compounds (i.e., crude extracts). Network pharmacology is an integrated in silico approach that can explain the modes of action of different drug leads or even complex natural-product-derived extracts by utilizing many bioinformatics-based databases and platforms. It also helps researchers to understand the complex interactions between drugs and their targets. Herein, we discovered that the EtOAc extract of the marine-derived soft coral *C. pachyclados* has the ability to inhibit the growth of two different BC cell lines (MCF7 and MDA-MA-231) with very good selectivity. Using this extract in the preparation of stable AgNPs made the anti-breast cancer effect of the final formulation very close to that of the reference anti-breast cancer agent, doxorubicin, with a much better selectivity index. This apparent improvement could be attributed to the ability of AgNPs to accumulate the extracts’ bioactive metabolites on their surface, making it much more potent (polyvalent effect).

A number of previously reported green synthesized AgNPs have shown interesting efficacy against human breast cancer cell lines (MCF-7 or MDA-MB-231), particularly those synthesized using plant extracts. For example, the green synthesis of AgNPs using *Lonicera hypoglauca* flower has been proven to induce apoptosis in MCF-7 cell lines [93]. Moreover, green synthesized AgNPs using *Alternanthera sessilis* extract have shown in vitro cytotoxicity against MCF-7 comparable to that of cisplatin [94]. With regard to MDA-MB-231, albumin-coated AgNPs have shown good activity against this type of breast cancer cells both in vitro and in vivo [95]. Soft corals are poorly studied as possible agents in the green synthesis of AgNPs, and hence, the results presented herein open the door for further investigations in this regard.

To understand the possible mode(s) of action of this bioactive extract, we utilized network pharmacology and other in silico approaches to comprehensively outline: (i) the main bioactive molecules in this extract; (ii) the most probable targets and signaling pathways that this extract could influence. First, the main chemical constituents in the extract were annotated with the aid of LC-HRESIMS chemical profiling. Secondly, these dereplicated compounds were subjected to two independent machine-learning-based prediction software platforms to highlight the most probable metabolites involved in the anti-breast cancer activity of the extract. Subsequently, the molecular targets of these bioactive metabolites were determined as follows: (i) collecting all genes or proteins reported to be linked to BC, using two independent databases to complete this step; (ii) subjecting the identified bioactive compounds to inverse docking against all proteins hosted in the PDB. In the light of the collected BC-related molecular targets, a PPI network for BC was constructed, and from this, the most important targets and signaling pathways relating to BC were outlined. Moreover, the BC-related targets for the main bioactive compounds in the extract could be extracted from the docking results. With the aid of this information and KEGG enrichment analysis, we constructed protein-pathway and compound-protein interaction networks to discover the most important signaling pathways influenced by the extract compounds and to determine the most highly interacting (i.e., important) compounds. Further validation of our results was conducted with the aid of MDS and Δ*G* calculations. As a result, compounds **5**–**9** and **12** were suggested, with a high level of confidence, to target six BC-related molecular targets. The molecular interactions between these compounds and their corresponding protein targets were also explained.

Network pharmacology has previously been utilized in the study of the anticancer properties of several natural products against breast cancer and found to be useful in the determination of the main active constituents in complex crude extracts [96,97,98]. For example, the anti-breast cancer mode of action and the potential targets of *Caesalpinia pulcherima* extract were determined and validated by the aid of network pharmacology approaches [99]. To the best of our knowledge, this is the first network-pharmacology-based study that integrates multiple in silico approaches with molecular dynamics simulation and Δ*G* determination to determine the probable anti-breast cancer active components. Additionally, it is the first comprehensive study that investigates the molecular targets of both MCF-7 and MDA-MB-231 at the same time.

With regard to the limitations of the present study, the cellular toxicity of AgNPs is among the main limitations. Previous in vitro toxicity studies on AgNPs have revealed that the dissolution of silver ions was the main cause of the observed cellular toxicity (i.e., mitochondrial toxicity). However, such toxicity appears to be size-dependent and associated with large doses of AgNPs [17,100,101]. In general, AgNPs induce lower toxicity against normal cell lines compared to cancerous ones, particularly if ordinary reducing agents were used for their preparation [12,102,103]. However, the phytosynthesis of AgNPs always leads them to acquire enhanced bioactivity, notably anticancer activity [104,105]. The development of silver-ion sequestering agents to be incorporated with the AgNPs may help in reducing such induced toxicity.

Although AgNPs are increasingly used in biological and pharmacological disciplines, there has been relatively little research on these in clinical medicine. This could be due to the previously discussed toxicity and the heterogeneity of the produced particles in terms of shape and size. Herein, our produced AgNPs were of various sizes and shapes. Moreover, they adsorbed a complex mixture of different compounds. According to our network pharmacology and in silico analyses, CE can kill BC cells via various mechanisms (e.g., necrosis, apoptosis, and autophagy) affecting multiple signaling pathways. Hence, evaluating the pharmacokinetics and the exact mode of action of the whole formulation is not straightforward.

Our future plans regarding this anti-breast cancer AgNP formulation include developing efficient and biocompatible silver-ion sequestering agents to overcome the possible long-term toxicity that could be produced. Additionally, we intend to work on optimizing the current preparation method so that we can produce nanoparticles with the highest possible homogeneity. Furthermore, depending on the network pharmacology results, we will isolate the most active compound(s) to be used instead of the whole extract in the AgNP formulation. Another option is to use these compounds as marker molecules in the extracts to make it much easier to evaluate the pharmacokinetics and pharmacodynamics of the whole formulation.

## 5. Conclusions

In conclusion, this natural-product-based AgNP formulation is a novel anti-breast cancer agent with an efficacy comparable to currently used chemotherapeutic agents. Hence, it is worth further developing a more homogenous and well-characterized formulation that can be evaluated in vivo. Additionally, the cheminformatics- and bioinformatics-derived information in this study can help in the future exploration of novel bioactive anti-breast cancer chemical entities from *C. pachyclados* and other under-explored soft corals.

## Figures and Tables

**Table 1:** Anticancer activity of CE and AgNPs against breast cancer and normal cell lines.

	MCF7 (SI)	MDA-MB-231 (SI)	MCF10a
CE	24.32 ± 0.52 ^c^ (2.95)	9.55 ± 0.53 ^b^ (7.52)	71.85 ± 0.5 ^c^
AgNPs	5.62 ± 0.26 ^b^ (7.34)	1.72 ± 0.14 ^a^ (24)	41.29 ± 0.44 ^b^
Doxorubicin	2.61 ± 0.03 ^a^ (7.7)	1.5 ± 0.26 ^a^ (13.4)	20.09 ± 0.72 ^a^

Selectivity index (SI) = IC_50_ of tested material in a normal cell line/IC_50_ of the material in cancer cell line, where IC_50_ is the concentration required to kill 50% of the cell population. Different letters indicate significant differences from repeated experiments (*n* = 3).

**Table 2:** Major compounds **1**–**20** (peak area > 10,000) that were annotated in the CE.

No.	Rt	*m*/*z*	Ionization Mode	Accurate Mass	Calculated Mass	Molecular Formula	Putative Identification	Chemical Class
**1**	9.11	431.3522	Positive	430.3449	430.3447	C_28_H_46_O_3_	(1) (24S)-3-β-Hydroxyergost-5-en-21-oic acid	Sterol
**2**	9.43	475.3426	Positive	474.3353	474.3345	C_29_H_46_O_5_	Hirsutosterol G	Sterol
**3**	9.15	299.2373	Positive	298.2395	298.2297	C_21_H_30_O	Pregn-1,20-dien-3-one	Sterol
**4**	9.28	297.2214	Positive	296.2141	296.2140	C_21_H_28_O	Pregn-1,4,20-trien-3-one	Sterol
**5**	8.88	315.2322	Negative	316.2395	316.2402	C_21_H_32_O_2_	Krempene C	Sterol
**6**	8.95	313.2165	Negative	314.2238	314.2246	C_21_H30O_2_	Krempene D	Sterol
**7**	5.43	463.2693	Positive	462.262	462.2618	C_26_H_38_O_7_	Krempfielin H	Diterpene (eunicellin derivative)
**8**	5.67	451.2698	Positive	450.2625	450.2618	C_25_H_38_O_7_	Krempfielin P	Diterpene (eunicellin derivative)
**9**	6.32	467.3005	Positive	466.2932	466.2931	C_26_H_42_O_7_	Australin C	Diterpene (eunicellin derivative)
**10**	5.78	321.2433	Positive	320.236	320.2351	C_20_H_32_O_3_	Cladiellisin	Diterpene (eunicellin derivative)
**11**	6.14	365.1962	Negative	366.2035	366.2042	C_20_H_30_O_6_	Hirsutocoquinone A	Diterpene (tocopherol derivative)
**12**	4.72	381.2642	Negative	382.2715	382.2719	C_22_H_38_O_5_	6-α-hydroxypolyanthelline A	Diterpene (eunicellin derivative)
**13**	7.83	363.2532	Positive	362.2459	362.2457	C_22_H_34_O_4_	Flaccidoxide	Diterpene (cembrane derivative)
**14**	8.17	405.2643	Positive	404.257	404.2563	C_24_H_36_O_5_	Flaccidoxide-13-acetate	Diterpene (cembrane derivative)
**15**	3.16	237.1857	Negative	238.193	238.1933	C_15_H_26_O_2_	Cladidiol	Sesquiterpene
**16**	3.74	250.2174	Positive	249.2101	249.2093	C_16_H_27_NO	Cladioxazole	Sesquiterpene (alkaloid)
**17**	3.89	269.0771	Negative	270.0844	270.0852	C_11_H_14_N_2_O_6_	5-O-Acetylthymidine	Nucleoside
**18**	3.65	259.1238	Negative	260.1311	260.1313	C_18_H_16_N_2_	Vibrindole A *	Indole alkaloid
**19**	2.98	223.9344	Negative	224.9417	224.9425	C_8_H_4_BrNO_2_	6-bromoisatin *	Indole alkaloid
**20**	2.56	135.0813	Positive	134.074	134.0732	C_9_H_10_O	*p*-Vinylbenzyl alcohol	Aromatic alcohol

* Identity further confirmed by comparison with authentic standards.

## Data Availability

Not applicable.

## Supplementary Materials

The following are available online at https://www.mdpi.com/article/10.3390/pharmaceutics13111846/s1. Figures S1 and S2: LC-HRESIMS base peak intensity chromatograms of CE, Figure S3: SAED images for as-prepared CE-AgNPs, Figure S4: KEGG-derived signaling Pathways in cancer. Red-colored numbers and arrows indicate CE-derived metabolites that were predicted to target a number of BC-related proteins, Figure S5: Dose-response curves of CE, AgNPs, and Doxorubicin against MCF7 (A–C), MDA-MB-231 (D–F), and MCF10a (G–I), respectively, Figure S6: MTT cytotoxicity assay results of CE, AgNPs, and Doxorubicin against MCF7 (A–C), MDA-MB-231 (D–F), and MCF10a (G–I), respectively. These histograms shows the absorbance of MTT -stained cells upon their treatment by various concentrations of CE, AgNPs, and Doxorubicin, Figure S7: AgNPs stability monitored by determination of surface plasmon band (SPB) after (5, 30, 60 days), Table S1: All collected BC-related targets (242 targets). Yellow-highlighted targets were collected from literature, while the other were collected from databases, Table S2: BC-related targets that were predicted to be inhibited by CE-derived compounds, Table S3: KEGG signaling pathway enrichment.
