# Supplementary material for: Anticancer Potential of Green Synthesized Silver Nanoparticles of the Soft Coral Cladiella pachyclados Supported by Network Pharmacology and In Silico Analyses
Source: Pharmaceutics. 2021 Nov 3;13(11):1846. doi: 10.3390/pharmaceutics13111846 (PMC8621232; doi:10.3390/pharmaceutics13111846)
Supplement: Supplementary file 1 [file pharmaceutics-13-01846-s001.zip › pharmaceutics-1379354-supplementary.pdf]

pharmaceutics

MDPI

Supplementary Materials: Anticancer Potential of Green Synthesized Silver Nanoparticles of the Soft Coral *Cladiella pachyclados* Supported by Network Pharmacology and In Silico Analyses

Hani A. Alhadrami, Heba Alkhatabi, Fahad H. Abduljabbar, Usama Ramadan Abdelmohsen and Ahmed M. Sayed

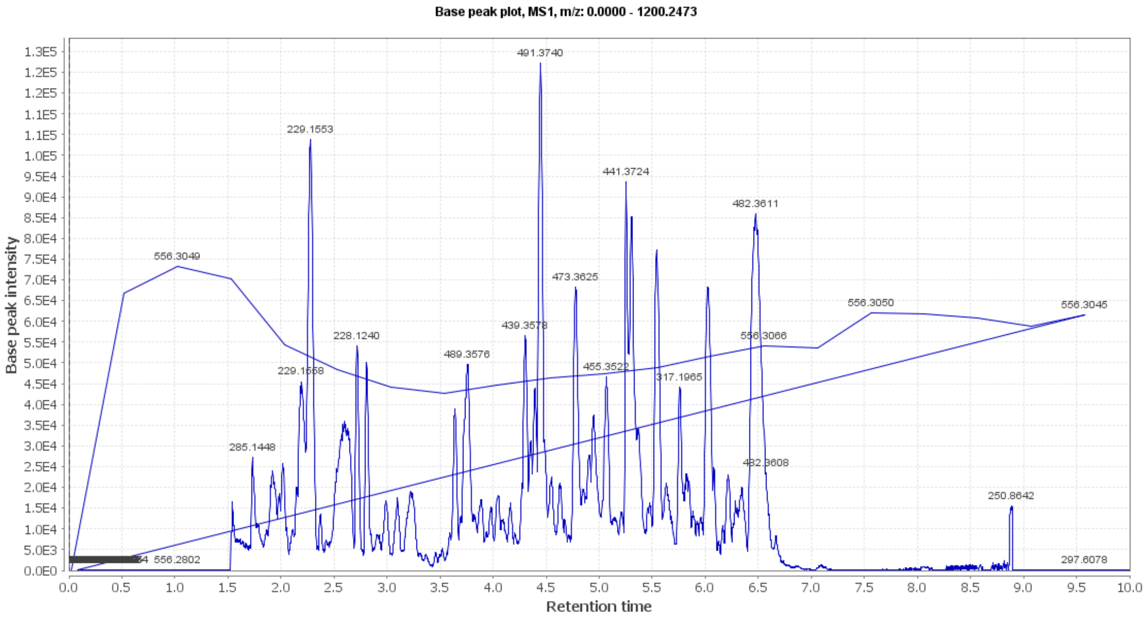

Figure S1. LC-HRESIMS base peak intensity (positive mode) chromatogram of CE.

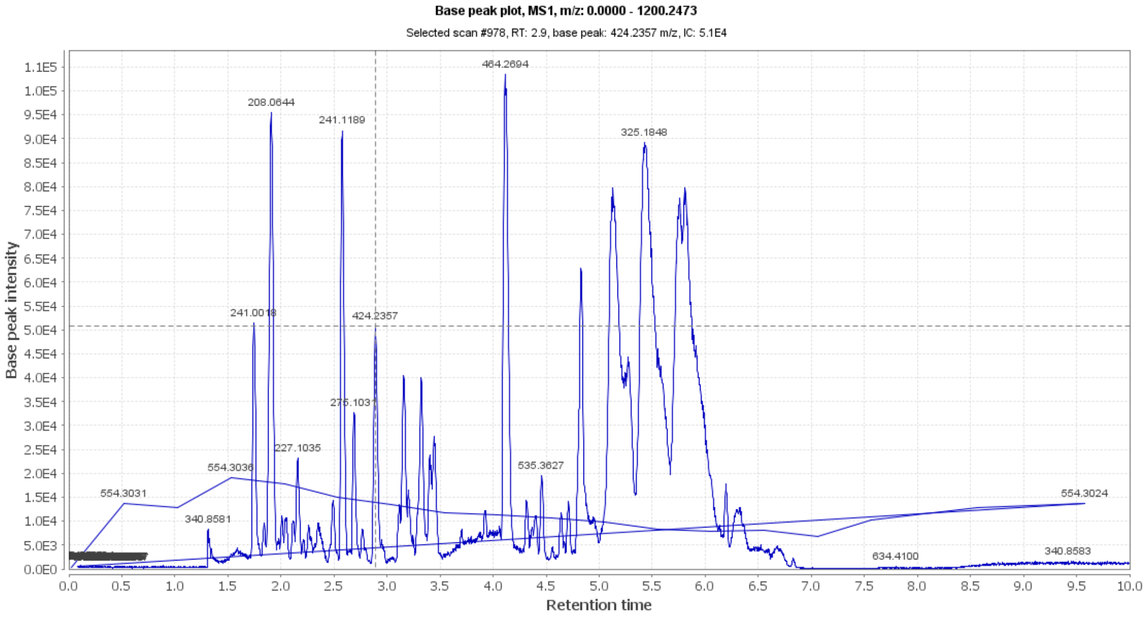

Figure S2. LC-HRESIMS base peak intensity (negative mode) chromatogram of CE.

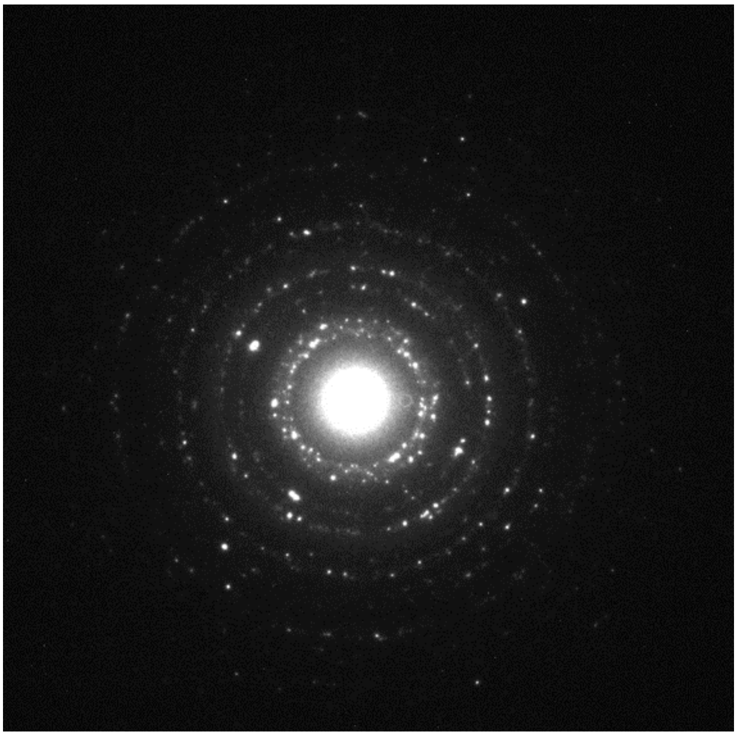

Figure S3. SAED images for as-prepared CE-AgNPs.

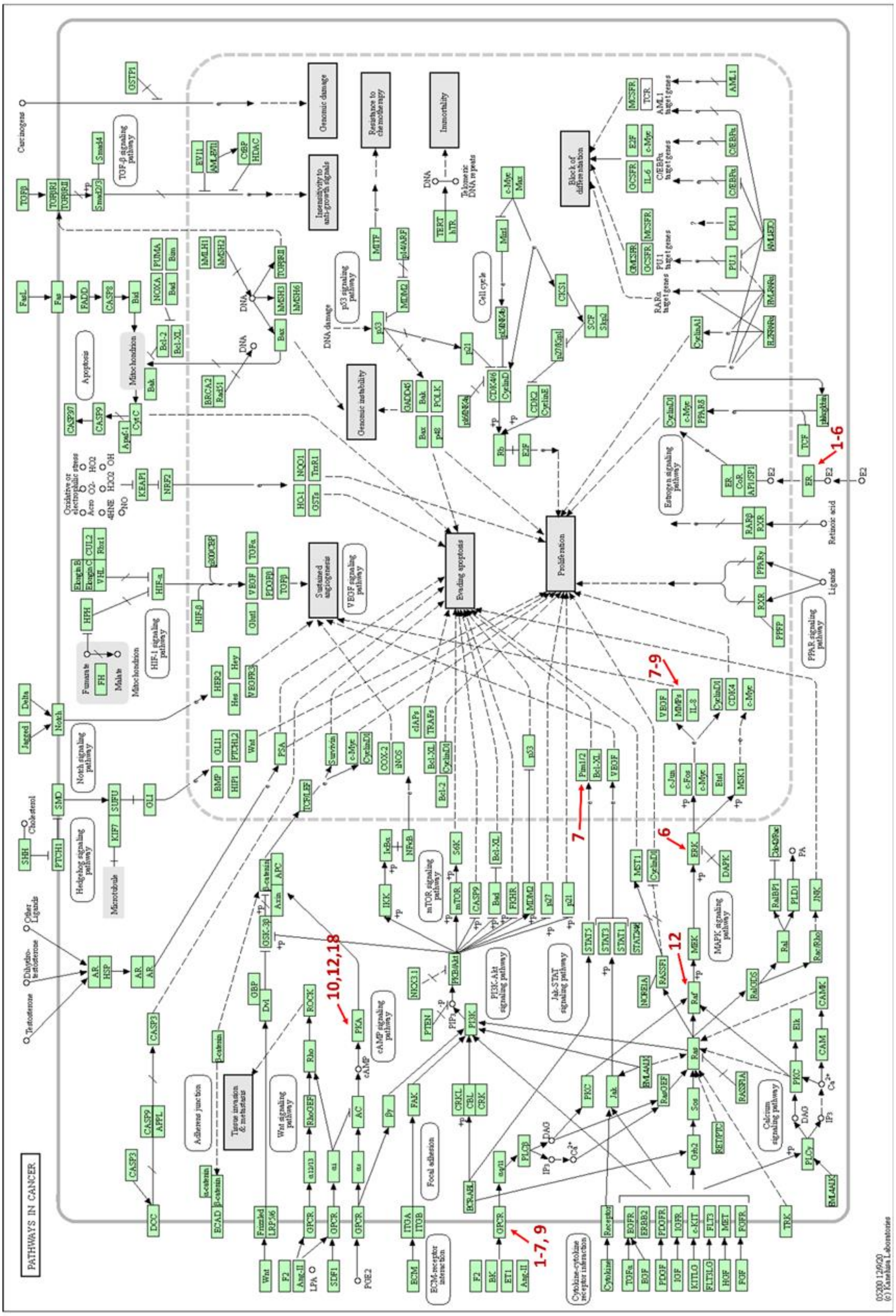

Figure S4. KEGG-derived signaling Pathways in cancer. Red-colored numbers and arrows indicate CE-derived metabolites that were predicted to target a number of BC-related proteins

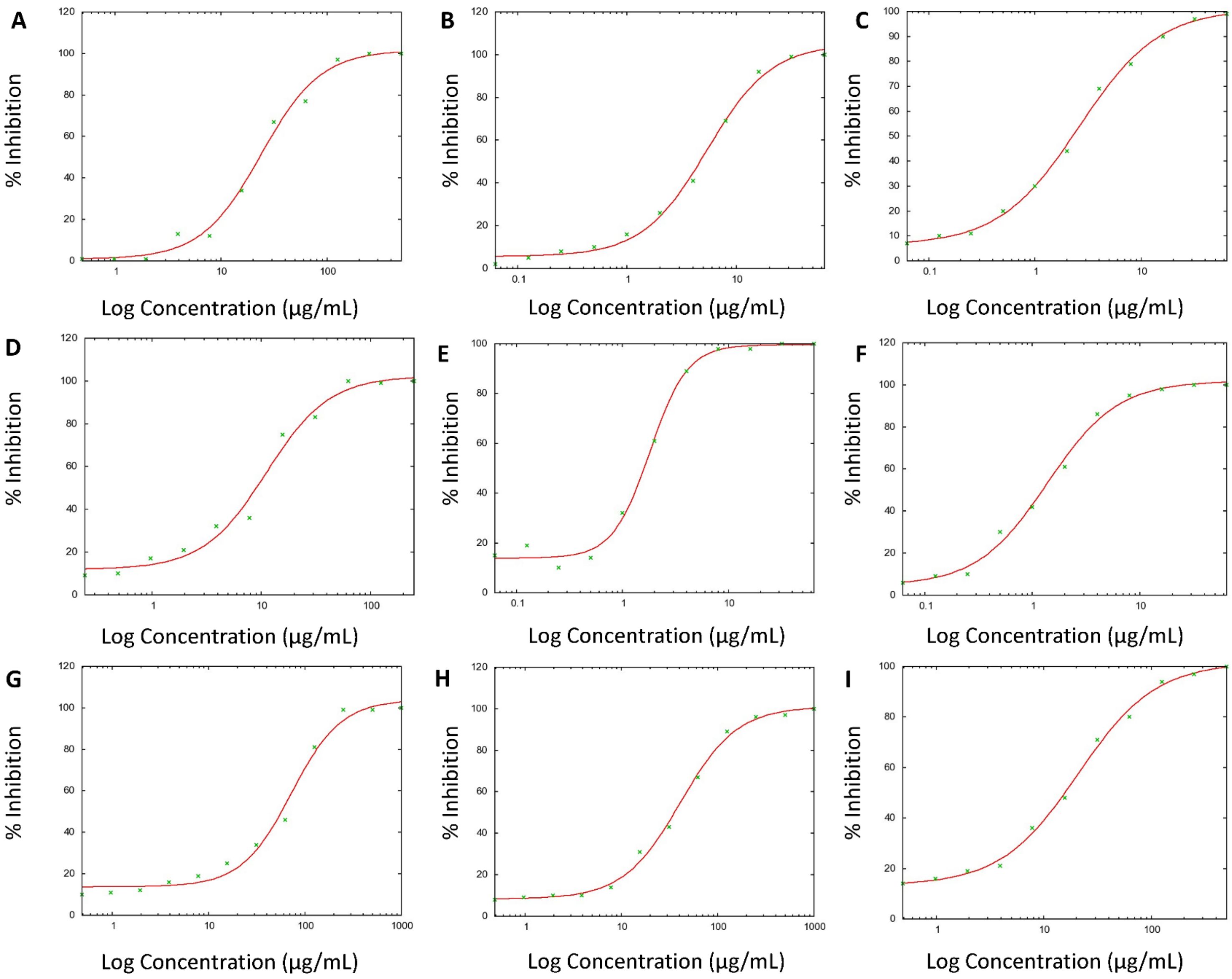

Figure S5. Dose-response curves of CE, AgNPs, and Doxorubicin against MCF7 (A-C), MDA-MB-231 (D-F), and MCF10a (G-I), respectively.

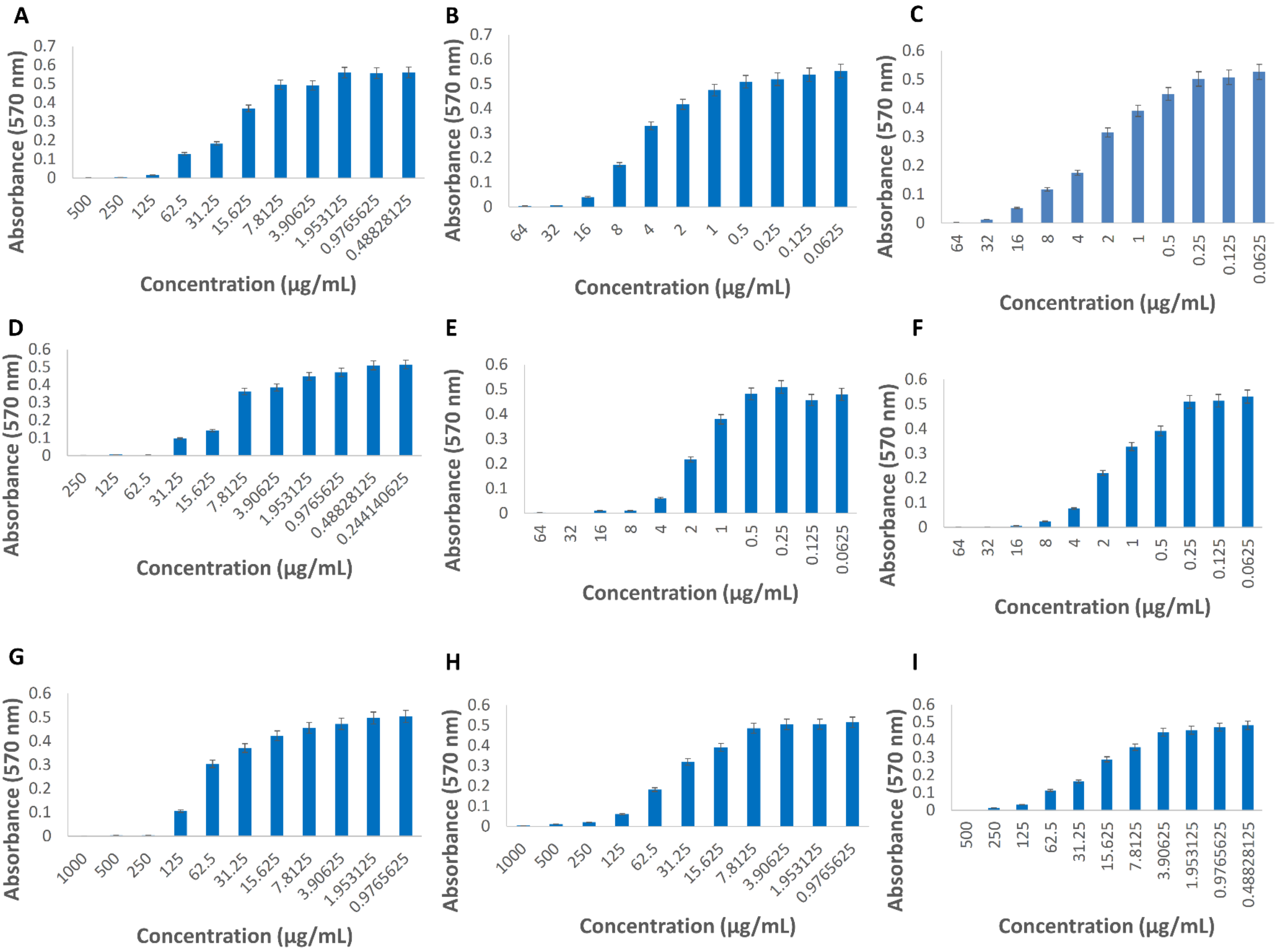

Figure S6. MTT cytotoxicity assay results of CE, AgNPs, and Doxorubicin against MCF7 (A-C), MDA-MB-231 (D-F), and MCF10a (G-I), respectively. These histograms shows the absorbance of MTT -stained cells upon their treatment by various concentrations of CE, AgNPs, and Doxorubicin.

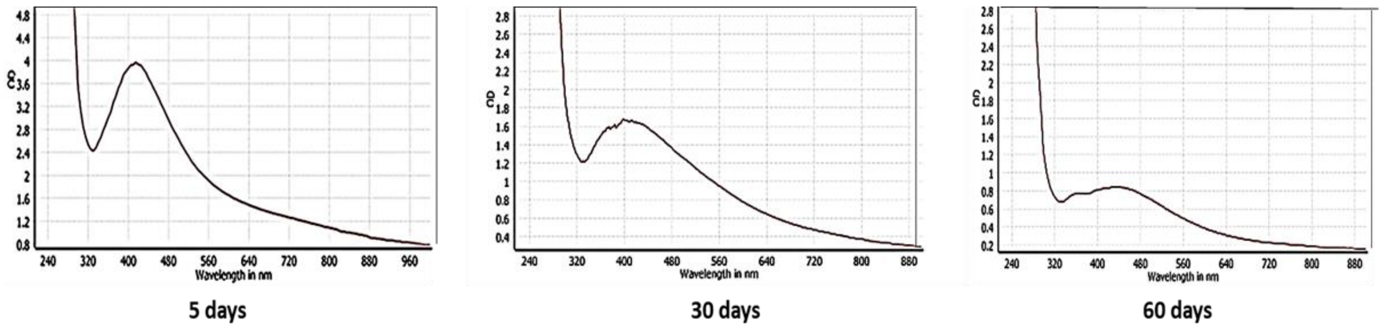

Figure S7. AgNPs stability monitored by determination of surface plasmon band (SPB) after (5, 30, 60 days).

Table S1. All collected BC-related targets (242 targets).

Target Abbreviation						
MAPK3	FYN	CYP19A1	BCAT1	CDKN2B	MYC	TAB1,
EP300	RARG	ALOX5	COL13A1a	CEBPB	NFKB1,	TAB2,
SRC	AKR1C3	MMP8	EXT1	CFLAR	NRAS,	TCF7
RXRA	RARB	ALOX15	LIMCH1a	CHEK1	PDGFB,	TFRC,
ADRA2A	NR3C1	ALOX12	AKR1B1a	CREB3L2	PDGFC,	TGFB1,
ADRA2C	ARG1	TACR1	CNN3a	CSF1R	PDGFRB,	TGFBR1,
ADRA2B	PTPN6	PTPN1	CYBRD1	CTNND1	PDK1,	TGFBR2,
PRKCD	PRKCH	AURKB	FXYD5	CX3CL1	PGK1,	THBS1,
VEGFA	ELANE	CDC25A	SRGNa	CXCL10	PIK3CA,	TIMP1,
DRD2	NOS3	CDC25B	IFI16a	CXCL11	PIM1,	TNF,
CNR1	PPARA	LDHA	IGFBP7a	CXCL16	PLA2G4A,	TNFRSF1A,
MAPK14	PTK2	NR1H2	TMEM158	CXCL8	PLK1	TNFRSF1B,
DRD3	FABP5	ALB	GNG11a	CXCL9	PMAIP1,	TRAF2,
OPRM1	IGF1R	TYMS	PLAC8a	DDIT3	PRKX,	VEGF
GABBR1	MDM2	PTGES	SRPX	DDIT4	PTGS2,	
ADORA3	PPARG	HDAC6	AGPS	EGFR	PTK2	
CNR2	FDFT1	MMP2	PTRF	EIF4EBP1	PTPN11,	
MTNR1A	BCL2	ESR2	ACKR3,	ETS1	PTPRF,	
MTNR1B	PTGS2	PPARD	BAMBI,	GAPDH	RAF1	
PTGER3	CDK2	CRHR1	BIRC5,	GRB2	RALBP1,	
NPY5R	MPO	PTGER2	BNIP3,	GSK3B	RASGRP2,	
CCR1	MMP9	PTGER4	BRAF,	GTSE1	RASSF1,	
HCAR2	NR1H3	NEK2	CCL13,	HIF1A	RASSF5,	
RXRB	PTGS1	BCL2L1	CCL5,	HSPB1	RHEB,	

EGFR	SREBF2	KLK2	CCND1,	IGFBP3	RHOA,	
RXRG	MET	HDAC8	CCNG2,	INHBA	RPS6,	
PTPN11	VDR	TBXAS1	CCR2,	IRAK1	RRAS	
KAT2B	ADRB2	MMP1	CCR7,	IRF3	SER-PINB5,	
ESR1	TTR	CA9	CD19,	LAMTOR3	SER-PINE1,	
PRKCG	CYP2C19	PGF	CD27,	LTBP1	SIAH1,	
RARA	PTGDR	SERPINE1	CD70,	LYN	SLC2A1,	
CDK1	FABP4	TERT	CD86,	MAP2K1	SMO	
PRKCB	AR	PIN1	CDC25B,	MAP2K2	SPI1,	
LCK	FABP1	RORA	CDC42,	MAP3K13	SPP1	
PRKCE	PTPN2	MCL1	CDH1,	MAX	SRC	
KDR	NOS2	HSD11B1	CDK2,	MET	STAT1,	
PRKCQ	FUCA1	PLA2G1B	CDK4,	MLLT4	STMN1,	
HIF1A	TOP2A	ADORA2B	CDKN2A,	MRAS	SYK	

Table S2. BC-related targets that were predicted to be inhibited by CE-derived compounds.

Target abbreviation	Target name	PDB code	Targeted by	Hub Protein
ERK1	MAP kinase ERK1	4QTB	6 (validated by MDS)	Yes
PRKCD	Protein kinase C delta	1PTR	9, 12	No
PTGER3	Prostaglandin E2 receptor EP3 subtype	6AK3	1-6	No
ESR1	Estrogen receptor	1SJ0	1-6	Yes
PRKCQ	Protein kinase C theta	1XJD	9	No
NR3C1	Glucocorticoid receptor	6EL9	3, 5	Yes
MMP9	Matrix metalloproteinase-9	1JKC	7-9 (validated by MDS)	No
PTGS1	Prostaglandin G/H synthase 1	6Y3C	1-3	No
CYP2C19	Cytochrome P450 2C19	4GQS	1-4	No
CYP19A1	Cytochrome P450 19A1 (Aromatase)	3S79	3-6 (5, 6 were validated by MDS)	No
ALOX15	Arachidonate 15-lipoxygenase	4NRE	18	No
CDC25B	M-phase inducer phosphatase 2	4WH7	2, 5	No
PTGES	Prostaglandin E synthase	3DWW	1	No
MMP2	72 kDa type IV collagenase	1HOV	7 (validated by MDS)	No
ESR2	Estrogen receptor beta	3OLS	1, 5, 6	No
MMP1	Interstitial collagenase	1CGE	7, 8 (validated by MDS)	No
BCAT1	Branched-chain-amino-acid aminotransferase, cytosolic	2COI	8, 16	No

PIM1	Serine/threonine-protein kinase PIM1	5C1Q	7	No
PTGS2	Cyclooxygenase-2	6BL4	9	No
RAF1	RAF proto-oncogene serine/threonine-protein kinase	1C1Y	12	No
RRAS	Ras-related protein R-Ras	2FN4	12, 18	No
SLC2A1	Glucose transporter	5C65	7, 9	No
SRC	Proto-oncogene tyrosine-protein kinase Src	1A07	12, 18	Yes
PLK1	Serine/threonine-protein kinase PLK1	1UMW	12, 18 (12 was validated by MDS)	No
PTK2	Focal adhesion kinase 1	1K04	11, 12, 18	Yes
CDK1	Cyclin-dependent kinase 1	6GU6	12, 18	Yes

Table S3. KEGG signaling pathway enrichment.

Description	Number of genes	FDR value	Genes
**Pathways in cancer**	**12**	**2.9E-10**	MMP2 ǀ RAF1 ǀ MAPK3 ǀ MMP1 ǀ PTK2 ǀ ESR2 ǀ PTGER3 ǀ PTGS2 ǀ MMP9 ǀ PIM1 ǀ ESR1 ǀ SLC2A1
**Estrogen signaling pathway**	**8**	**1.25E-09**	MMP2 ǀ RAF1 ǀ MAPK3 ǀ ESR2 ǀ MMP9 ǀ SRC ǀ PRKCD ǀ ESR1
**Proteoglycans in cancer**	**8**	**1.45E-08**	MMP2 ǀ RRAS ǀ RAF1 ǀ MAPK3 ǀ PTK2 ǀ MMP9 ǀ SRC ǀ ESR1
**C-type lectin receptor signaling pathway**	**6**	**3.88E-07**	RRAS ǀ RAF1 ǀ MAPK3 ǀ PTGS2 ǀ SRC ǀ PRKCD
**MicroRNAs in cancer**	**6**	**2.56E-06**	RAF1 ǀ MAPK3 ǀ PTK2 ǀ PTGS2 ǀ SRC
**VEGF signaling pathway**	**5**	**9.55E-07**	PTGES ǀ PTGS1 ǀ PTGS2 ǀ CYP2C19 ǀ ALOX15
**Arachidonic acid metabolism**	**5**	**1.05E-06**	CDC25B ǀ RAF1 ǀ MAPK3 ǀ PTGS2 ǀ MMP9 ǀ PIM1
**Autophagy - animal**	**5**	**2.25E-05**	RRAS ǀ RAF1 ǀ MAPK3 ǀ PRKCQ ǀ PRKCD
**Chemokine signaling pathway**	**5**	**0.00011**	RAF1 ǀ MAPK3 ǀ PTK2 ǀ SRC ǀ PRKCD
**Regulation of actin cytoskeleton**	**5**	**0.00016**	MAPK3 ǀ MMP1 ǀ PTGS2 ǀ MMP9
**IL-17 signaling pathway**	**4**	**0.00015**	RRAS ǀ RAF1 ǀ MAPK3 ǀ PTK2 ǀ SRC
**AGE-RAGE signaling pathway in diabetic complications**	**4**	**0.00018**	MMP2 ǀ MAPK3 ǀ PIM1 ǀ PRKCD
**Breast cancer**	**4**	**0.00068**	RAF1 ǀ MAPK3 ǀ ESR2 ǀ ESR1
**Cellular senescence**	**4**	**0.00073**	RRAS ǀ RAF1 ǀ MAPK3 ǀ CDK1
**Focal adhesion**	**4**	**0.0016**	RAF1 ǀ MAPK3 ǀ SLC2A1
**Rap1 signaling pathway**	**4**	**0.0017**	RAF1 ǀ MAPK3 ǀ PTK2 ǀ SRC
**cAMP signaling pathway**	**4**	**0.0018**	RRAS ǀ RAF1 ǀ MAPK3 ǀ SRC
**MAPK signaling pathway**	**4**	**0.0048**	RAF1 ǀ MAPK3 ǀ SRC
**Central carbon metabolism in cancer**	**3**	**0.0014**	RRAS ǀ RAF1 ǀ MAPK3 ǀ PTGER3
**EGFR tyrosine kinase inhibitor resistance**	**3**	**0.0018**	PRKCQ ǀ SRC ǀ PRKCD
**Inflammatory mediator regulation of TRP channels**	**3**	**0.0028**	MAPK3 ǀ PTGS2 ǀ MMP9

**TNF signaling pathway**	**3**	**0.0042**	CDC25B丨RRAS丨RAF1丨MAPK3
**Cell cycle**	**3**	**0.0048**	CDC25B丨PLK1丨CDK1
**FoxO signaling pathway**	**3**	**0.0052**	RAF1丨MAPK3丨PLK1
**Phospholipase D signaling pathway**	**3**	**0.0074**	CYP2C19丨ALOX15
**Ras signaling pathway**	**3**	**0.0214**	RRAS丨RAF1丨MAPK3
**Linoleic acid metabolism**	**2**	**0.0058**	RRAS丨RAF1丨MAPK3
**Adherens junction**	**2**	**0.0228**	MAPK3丨SRC
**Th1 and Th2 cell differentiation**	**2**	**0.0322**	MAPK3丨PRKCQ
**Choline metabolism in cancer**	**2**	**0.0375**	RAF1丨MAPK3
**NF-kappa B signaling pathway**	**2**	**0.0409**	PRKCQ丨PTGS2
**Th17 cell differentiation**	**2**	**0.0409**	MAPK3丨PRKCQ

In vitro cytotoxicity raw data

Cytotoxicity experiments were carried out according to the MTT assay protocol. After staining the viable cells with MTT, their absorbance were measured at 570 nm. The assays were repeated three times. The measured absorbance of each test material at different concentrations was then used in the following formula to calculate %inhibition:

$\%\text{inhibition} = 100 - (At/Ac) \times 100$

At = Absorbance value of test compound

Ac = Absorbance value of control, which was 0.566

The background absorbance (blank absorbance) was automatically subtracted by the machine.

The following data are: (i) absorbance of treated cells at different concentrations of the test material in three experiments (Figure S5 shows the average absorbance at different concentrations of the test material); (ii) the calculated %inhibitions for each tested concentration in each experiment. The IC50 was then calculated for each experiment and then the Mean IC50 ± SD was taken for each test material against the corresponding cell type.

1- Cytotoxicity raw data of CE against MCF7

inhibitor Concentration (µg/mL)	Absorbance		
	Experiment 1	Experiment 2	Experiment 3
500	0.001132	0.00283	0.005094
250	0.003396	0.003962	0.004528
125	0.015565	0.007188	0.004698
62.5	0.128539	0.156556	0.118011
31.25	0.183214	0.216099	0.178743
15.625	0.369485	0.349675	0.389521
7.8125	0.495929	0.459762	0.474365
3.90625	0.491005	0.481043	0.487722
1.953125	0.560057	0.550492	0.556718
0.976563	0.556887	0.557567	0.559265
0.488281	0.56068	0.561302	0.56

inhibitor Concentration (µg/mL)	%inhibition		
	Experiment 1	Experiment 2	Experiment 3
500	99.8	99.5	99.1
250	99.4	99.3	99.2
125	97.25	98.73	99.17
62.5	77.29	72.34	79.15
31.25	67.63	61.82	68.42
15.625	34.72	38.22	31.18
7.8125	12.38	18.77	16.19
3.90625	13.25	15.01	13.83
1.953125	1.05	2.74	1.64
0.9765625	1.61	1.49	1.19
0.48828125	0.94	0.83	1.06
IC50	23.83	24.86	24.27
Mean IC50	24.32 ± 0.52		

2- Cytotoxicity raw data of AgNPs against MCF7

inhibitor Concentration (µg/mL)	Absorbance		
	Experiment 1	Experiment 2	Experiment 3
64	0.003226	0.000623	0.001358
32	0.00549	0.001302	0.002038
16	0.040695	0.033734	0.006339
8	0.171838	0.151575	0.163687
4	0.329808	0.316281	0.326129
2	0.416972	0.445555	0.440405
1	0.474478	0.46961	0.480081
0.5	0.509004	0.50125	0.50623
0.25	0.519079	0.524795	0.519645
0.125	0.537247	0.536738	0.54053
0.0625	0.552473	0.544492	0.552246

inhibitor Concentration (µg/mL)	%inhibition		
	Experiment 1	Experiment 2	Experiment 3
64	99.43	99.89	99.76
32	99.03	99.77	99.64
16	92.81	94.04	98.88
8	69.64	73.22	71.08
4	41.73	44.12	42.38
2	26.33	21.28	22.19
1	16.17	17.03	15.18
0.5	10.07	11.44	10.56
0.25	8.29	7.28	8.19
0.125	5.08	5.17	4.5
0.0625	2.39	3.8	2.43
IC50	5.38	5.57	5.89
Mean IC50	5.62 ± 0.26		

3- Cytotoxicity raw data of Doxorubicin against MCF7

inhibitor Concentration (µg/mL)	Absorbance		
	Experiment 1	Experiment 2	Experiment 3
64	0.001472	0.004019	0.00583
32	0.012395	0.01115	0.015282
16	0.051846	0.044714	0.048789
8	0.117219	0.104427	0.093786
4	0.174328	0.15814	0.131312
2	0.315432	0.287528	0.344298
1	0.390597	0.389238	0.407124
0.5	0.449857	0.437858	0.423311
0.25	0.501872	0.452234	0.47561
0.125	0.507815	0.487043	0.502438
0.0625	0.526097	0.507362	0.512173

inhibitor Concentration (µg/mL)	%inhibition		
	Experiment 1	Experiment 2	Experiment 3
64	99.74	99.29	98.97
32	97.81	98.03	97.3
16	90.84	92.1	91.38
8	79.29	81.55	83.43
4	69.2	72.06	76.8
2	44.27	49.2	39.17
1	30.99	31.23	28.07
0.5	20.52	22.64	25.21
0.25	11.33	20.1	15.97
0.125	10.28	13.95	11.23
0.0625	7.05	10.36	9.51
IC50	2.59	2.65	2.61
Mean IC50	2.61 ± 0.03		

4- Cytotoxicity raw data of CE against MDA-MB-231

inhibitor Concentration (µg/mL)	Absorbance		
	Experiment 1	Experiment 2	Experiment 3
250	0.001698	0.002264	0.003396
125	0.00566	0.003962	0.004528
62.5	0.00283	0.00566	0.01132
31.25	0.09622	0.04528	0.13584
15.625	0.1415	0.09622	0.1132
7.8125	0.36224	0.29432	0.35092
3.90625	0.38488	0.38488	0.34526
1.953125	0.44714	0.45846	0.51506
0.976563	0.46978	0.45846	0.4528
0.488281	0.5094	0.50374	0.49808
0.244141	0.51506	0.49808	0.5094

inhibitor Concentration (µg/mL)	%inhibition		
	Experiment 1	Experiment 2	Experiment 3
250	99.71	99.63	99.42
125	99.32	99.38	99.2
62.5	99.5	99	98.63
31.25	83.27	92.19	76.23
15.625	75.57	83.11	80.8
7.8125	36.64	48.37	38.31
3.90625	32.03	32.4	39.28
1.953125	21.19	19.02	9.98
0.9765625	17.94	19.54	20.1
0.48828125	10.06	11.37	12.28
0.244140625	9.18	12.3	10.45
IC50	10.17	9.182	9.316
Mean IC50	9.55 ± 0.53		

5- Cytotoxicity raw data of AgNPs against MDA-MB-231

inhibitor Concentration (µg/mL)	Absorbance		
	Experiment 1	Experiment 2	Experiment 3
64	0.002943	0.000283	0.004132
32	0.001245	0.009226	0.004585
16	0.010245	0.002547	0.001868
8	0.011037	0.004188	0.009735
4	0.061468	0.063166	0.083825
2	0.217004	0.207835	0.252493
1	0.37956	0.318092	0.31679
0.5	0.481496	0.453592	0.442442
0.25	0.509343	0.482515	0.519192
0.125	0.456932	0.459762	0.506457
0.0625	0.479515	0.425858	0.520494

inhibitor Concentration (µg/mL)	%inhibition		
	Experiment 1	Experiment 2	Experiment 3
64	99.48	99.95	99.27
32	99.78	98.37	99.19
16	98.19	99.55	99.67
8	98.05	99.26	98.28
4	89.14	88.84	85.19
2	61.66	63.28	55.39
1	32.94	43.8	44.03
0.5	14.93	19.86	21.83
0.25	10.01	14.75	8.27
0.125	19.27	18.77	10.52
0.0625	15.28	24.76	8.04
IC50	1.81	1.56	1.79
Mean IC50	1.72 ± 0.14		

6- Cytotoxicity raw data of Doxorubicin against MDA-MB-231

inhibitor Concentration (μg/mL)	Absorbance		
	Experiment 1	Experiment 2	Experiment 3
64	0.000566	0.001868	0.000736
32	0.00034	0.001075	0.001641
16	0.006	0.004132	0.004641
8	0.024678	0.03945	0.036564
4	0.075278	0.139632	0.047035
2	0.220287	0.234098	0.246606
1	0.326978	0.364221	0.290132
0.5	0.391559	0.451328	0.421557
0.25	0.509174	0.500797	0.489647
0.125	0.513871	0.517324	0.541322
0.0625	0.530795	0.490779	0.504589

inhibitor Concentration (μg/mL)	%inhibition		
	Experiment 1	Experiment 2	Experiment 3
64	99.9	99.67	99.87
32	99.94	99.81	99.71
16	98.94	99.27	99.18
8	95.64	93.03	93.54
4	86.7	75.33	91.69
2	61.08	58.64	56.43
1	42.23	35.65	48.74
0.5	30.82	20.26	25.52
0.25	10.04	11.52	13.49
0.125	9.21	8.6	4.36
0.0625	6.22	13.29	10.85
IC50	1.37	1.81	1.34
Mean IC50	1.5 ± 0.26		

7- **Cytotoxicity raw data of CE against MCF10a**

inhibitor Concentration (µg/mL)	Absorbance		
	Experiment 1	Experiment 2	Experiment 3
1000	0.000623	0.000113	0.003113
500	0.004075	0.000396	0.006113
250	0.004188	0.010754	0.020659
125	0.105446	0.094126	0.074089
62.5	0.303942	0.288603	0.33128
31.25	0.370504	0.388389	0.378201
15.625	0.422293	0.396257	0.386465
7.8125	0.454894	0.454555	0.464573
3.90625	0.472553	0.484722	0.495986
1.953125	0.497005	0.487156	0.504872
0.976563	0.503627	0.505325	0.521739

inhibitor Concentration (µg/mL)	%inhibition		
	Experiment 1	Experiment 2	Experiment 3
1000	99.89	99.98	99.45
500	99.28	99.93	98.92
250	99.26	98.1	96.35
125	81.37	83.37	86.91
62.5	46.3	49.01	41.47
31.25	34.54	31.38	33.18
15.625	25.39	29.99	31.72
7.8125	19.63	19.69	17.92
3.90625	16.51	14.36	12.37
1.953125	12.19	13.93	10.8
0.9765625	11.02	10.72	7.82
IC50	72.38	71.39	71.78
Mean IC50	71.85 ± 0.5		

8- Cytotoxicity raw data of AgNPs against MCF10a

inhibitor Concentration (µg/mL)	Absorbance		
	Experiment 1	Experiment 2	Experiment 3
1000	0.004075	0.002094	0.000509
500	0.011433	0.009848	0.001472
250	0.020999	0.015961	0.004075
125	0.061015	0.07041	0.048393
62.5	0.182478	0.2234	0.195893
31.25	0.318771	0.298339	0.285547
15.625	0.390427	0.375484	0.351939
7.8125	0.485232	0.468139	0.45829
3.90625	0.504872	0.502268	0.484666
1.953125	0.505551	0.513588	0.499382
0.976563	0.514834	0.510928	0.505778

inhibitor Concentration (µg/mL)	%inhibition		
	Experiment 1	Experiment 2	Experiment 3
1000	99.28	99.63	99.91
500	97.98	98.26	99.74
250	96.29	97.18	99.28
125	89.22	87.56	91.45
62.5	67.76	60.53	65.39
31.25	43.68	47.29	49.55
15.625	31.02	33.66	37.82
7.8125	14.27	17.29	19.03
3.90625	10.8	11.26	14.37
1.953125	10.68	9.26	11.77
0.9765625	9.04	9.73	10.64
IC50	40.97	41.12	41.79
Mean IC50	41.29 ± 0.44		

9- Cytotoxicity raw data of Doxorubicin against MCF10a

inhibitor Concentration (µg/mL)	Absorbance		
	Experiment 1	Experiment 2	Experiment 3
500	0.00034	0.000509	0.000113
250	0.013471	0.008433	0.007188
125	0.031753	0.041941	0.050714
62.5	0.112577	0.094579	0.081051
31.25	0.16397	0.171894	0.144896
15.625	0.289056	0.312772	0.336034
7.8125	0.358278	0.390087	0.402879
3.90625	0.443574	0.42184	0.453253
1.953125	0.454328	0.470176	0.498023
0.976563	0.471818	0.479742	0.507249
0.488281	0.482685	0.497967	0.510362

inhibitor Concentration (µg/mL)	%inhibition		
	Experiment 1	Experiment 2	Experiment 3
500	99.94	99.91	99.98
250	97.62	98.51	98.73
125	94.39	92.59	91.04
62.5	80.11	83.29	85.68
31.25	71.03	69.63	74.4
15.625	48.93	44.74	40.63
7.8125	36.7	31.08	28.82
3.90625	21.63	25.47	19.92
1.953125	19.73	16.93	12.01
0.9765625	16.64	15.24	10.38
0.48828125	14.72	12.02	9.83
IC50	20.8	20.11	19.36
Mean IC50	20.09 ± 0.72		

Experimental

1. Chemical Profiling

The prepared crude extract was dissolved in methanol to reach a concentration of 1mg/mL for mass spectrometry analysis. An Acquity Ultra Performance Liquid Chromatography system attached to a Synapt G2 HDMS quadrupole time of flight hybrid mass spectrometer (Waters, Milford, the USA) was utilized. Positive and negative ESI ionization modes were employed to get out the high resolution mass spectrometry connected with a spray voltage at 4.5 kV, the capillary temperature at 320°C, and mass range from *m/z* 150–1500. The MS dataset was processed and data were obtained utilizing MZmine 2.20 based on the accepted parameters. Mass ion peaks were identified and accompanied by chromatogram builder and chromatogram deconvolution. The local minimum search algorithm was addressed and isotopes were too analyzed via the isotopic peaks of grouper. Missing peaks were displayed using the gap-filling peak finder. An adduct search along with a complex search was carried out. The processed data set was later exposed to molecular formula prediction and peak identification. The positive and negative ionization mode data sets from the respective extract were dereplicated against the DNP (Dictionary of Natural Products) databases.

2. Molecular Dynamics Simulation

Desmond v. 2.2 software was used for performing MDS experiments [1–3]. This software applies the OPLS force field. Protein systems were built using the System Builder option, where the protein structure was embedded in an orthorhombic box of TIP3P water together with 0.15 M Na^+ and Cl^- ions in 20 Å solvent buffer. Afterward, the prepared systems were energy minimized and equilibrated for 10 ns. Desmond software automatically parameterizes inputted ligands during the system building step according to the OPLS force field.

Metal-containing proteins like MMPs that contain histidine-Zn^{+2} complex in the active site should be parameterized during the protein preparation step. To do so, a hetero state should be generated for hetero atoms like Zn (Generate Hetero States). This function is a part of the maestro's Protein Preparation wizard. This step will enable the formation of a suitable hetero state or co-ordinate covalent state for the heteroatom (i.e. Zn^{+2}) in complex with the protein so that force fields like OPLS can easily recognize the zinc atom.

For simulations performed by NAMD [4], the parameters and topologies of the compounds were calculated either using the Charmm27 force field with the online software Ligand Reader and Modeler (http://www.charmm-gui. org/?doc=input/ligandrm, accessed on 16 April 2021) [5] or using the VMD plugin Force Field Toolkit (ffTK). Afterward, the generated parameters and topology files were loaded to VMD to readily read the protein–ligand complexes without errors and then conduct the simulation step. For MMPs, harmonic Tcl forces were applied to keep Zn^{+2} in place.

3. Binding Free Energy Calculations

Binding free energy calculations (ΔG) were performed using the free energy perturbation (FEP) method [4]. This method was described in detail in the recent article by Kim and coworkers [4]. Briefly, this method calculates the binding free energy $\Delta G_{binding}$ according to the following equation: $\Delta G_{binding} = \Delta G_{Complex} - \Delta G_{Ligand}$. The value of each ΔG is estimated from a separate simulation using NAMD software. Interestingly, all input files required for simulation by NAMD can be papered by using the online website Charmm-GUI (https://charmm-gui.org/?doc=input/afes.abinding, accessed on 18 May 2021). Subsequently, we can use these files in NAMD to produce the required simulations using the FEP calculation function in NAMD. The equilibration was achieved in the NPT ensemble at 300 K and 1 atm (1.01325 bar) with Langevin piston pressure (for "Complex" and "Ligand") in the presence of the TIP3P water model. Then, 10 ns FEP simulations were performed for each compound, and the last 5 ns of the free energy values was measured for

the final free energy values [4]. Finally, the generated trajectories were visualized and analyzed using VMD software. It worth noting that Ngo and co-workers in their recent benchmarking study found that the FEP method of determination of ΔG was the most accurate method in terms of predicting M^{Pro} inhibitors [5].

References

1. Bowers, K.J.; Chow, D.E.; Xu, H.; Dror, R.O.; Eastwood, M.P.; Gregersen, B.A.; Klepeis, J.L.; Kolossvary, I.; Moraes, M.A.; Sacerdoti, F.D.; et al. Scalable algorithms for molecular dynamics simulations on commodity clusters. In Proceedings of the SC'06: Proceedings of the 2006 ACM/IEEE Conference on Supercomputing, Tampa, FL, USA, 11–17 November 2006; IEEE: New York, NY, USA, 2006; p. 43.

2. Release, S. 3: Desmond Molecular Dynamics System, DE Shaw Research, New York, NY, 2017; Maestro-Desmond Interoperability Tools, Schrödinger: New York, NY, USA, 2017.

7. Schrodinger LLC. Maestro, Version 9.0; Schrodinger LLC: New York, NY, USA, 2009.

3. Phillips, J.C.; Braun, R.; Wang, W.; Gumbart, J.; Tajkhorshid, E.; Villa, E.; Chipot, C.; Skeel, R.D.; Kalé, L.; Schulten, K. Scalable molecular dynamics with NAMD. J. Comput. Chem. 2005, 26, 1781–1802.

4. Kim, S.; Oshima, H.; Zhang, H.; Kern, N.R.; Re, S.; Lee, J.; Rous, B.; Sugita, Y.; Jiang, W.; Im, W. CHARMM-GUI free energy calculator for absolute and relative ligand solvation and binding free energy simulations. J. Chem. Theory Comput. 2020, 16, 7207–7218.

5. Ngo, S.T.; Tam, N.M.; Quan, P.M.; Nguyen, T.H. Benchmark of Popular Free Energy Approaches Revealing the Inhibitors Binding to SARS-CoV-2 Mpro. J. Chem. Inf. Model. 2021, 61, 2302–2312.
